# Spatial multiomics map of trophoblast development in early pregnancy

**DOI:** 10.1038/s41586-023-05869-0

**Published:** 2023-03-29

**Authors:** Anna Arutyunyan, Kenny Roberts, Kevin Troulé, Frederick C. K. Wong, Megan A. Sheridan, Ilia Kats, Luz Garcia-Alonso, Britta Velten, Regina Hoo, Elias R. Ruiz-Morales, Carmen Sancho-Serra, Jarrod Shilts, Louis-Francois Handfield, Luca Marconato, Elizabeth Tuck, Lucy Gardner, Cecilia Icoresi Mazzeo, Qian Li, Iva Kelava, Gavin J. Wright, Elena Prigmore, Sarah A. Teichmann, Omer Ali Bayraktar, Ashley Moffett, Oliver Stegle, Margherita Y. Turco, Roser Vento-Tormo

**Affiliations:** 1 Wellcome Sanger Institute, Cambridge, UK; 2 Centre for Trophoblast Research, University of Cambridge, Cambridge, UK; 3 Department of Pathology, University of Cambridge, Cambridge, UK; 4 Division of Computational Genomics and Systems Genetics, German Cancer Research Center (DKFZ), Heidelberg, Germany; 5 European Molecular Biology Laboratory, Genome Biology Unit, Heidelberg, Germany; 6 Department of Biology, Hull York Medical School, York Biomedical Research Institute, University of York, York, UK; 7 Theory of Condensed Matter, Cavendish Laboratory, Department of Physics, University of Cambridge, Cambridge, UK; 8 Present Address: Friedrich Miescher Institute for Biomedical Research, Basel, Switzerland

**Keywords:** Development, Biotechnology

## Abstract

The relationship between the human placenta—the extraembryonic organ made by the fetus, and the decidua—the mucosal layer of the uterus, is essential to nurture and protect the fetus during pregnancy. Extravillous trophoblast cells (EVTs) derived from placental villi infiltrate the decidua, transforming the maternal arteries into high-conductance vessels^1^. Defects in trophoblast invasion and arterial transformation established during early pregnancy underlie common pregnancy disorders such as pre-eclampsia^2^. Here we have generated a spatially resolved multiomics single-cell atlas of the entire human maternal–fetal interface including the myometrium, which enables us to resolve the full trajectory of trophoblast differentiation. We have used this cellular map to infer the possible transcription factors mediating EVT invasion and show that they are preserved in in vitro models of EVT differentiation from primary trophoblast organoids^3,4^ and trophoblast stem cells^5^. We define the transcriptomes of the final cell states of trophoblast invasion: placental bed giant cells (fused multinucleated EVTs) and endovascular EVTs (which form plugs inside the maternal arteries). We predict the cell–cell communication events contributing to trophoblast invasion and placental bed giant cell formation, and model the dual role of interstitial EVTs and endovascular EVTs in mediating arterial transformation during early pregnancy. Together, our data provide a comprehensive analysis of postimplantation trophoblast differentiation that can be used to inform the design of experimental models of the human placenta in early pregnancy.

## Abstract (web summary)

A multiomics single-cell atlas of the human maternal–fetal interface including the myometrium, combining spatial transcriptomics data with chromatin accessibility, provides a comprehensive analysis of cell states as placental cells infiltrate the uterus during early pregnancy.

## Main

During the nine months of human pregnancy, the successful development of the fetus is entirely dependent on its placenta. This transient extraembryonic organ is located at the interface between the mother and her fetus. Placental trophoblast cells arise from the trophectoderm surrounding the preimplantation embryo^6^. After implantation, EVTs emerge from the cytotrophoblast shell, infiltrate the decidua—the mucosal lining of the pregnant uterus, and migrate towards the spiral arteries where they destroy the smooth muscle media. Subsequently, endovascular trophoblast cells (eEVTs) form a plug close to the cytotrophoblast shell where the arteries terminate and then eEVTs replace the endothelium^1^. In this way EVTs transform maternal arteries in the decidua basalis into high-conductance vessels^2,7–9^. EVTs fuse into placental bed giant cells (GCs) around the decidual–myometrial boundary and normally invade only as far as the inner third of the myometrium^10^. Placentation and successful pregnancy depend on the correct degree of trophoblast invasion, and the decidua has an important role in this process^11,12^.

Our previous single-cell transcriptomics analysis of the first trimester maternal–fetal interface provided an unprecedented view of the cell states comprising this environment^13^. However, trophoblast cells present in the deeper layers of the decidua and myometrium are only present in samples of pregnant hysterectomies, and the villous syncytiotrophoblast (SCT), a multinucleated layer, is lost in classical single-cell RNA sequencing (scRNA-seq). A further difficulty is the loss of spatial context in these samples, which is essential to systematically resolve the interactions between trophoblast and decidual cells in early pregnancy. In addition, novel in vitro models have been developed recently, including trophoblast stem cells (TSCs) expanded in vitro^5^ and self-renewing primary trophoblast organoids^3,4,14^ (PTOs). These models can recapitulate some aspects of placental development and invasion, opening paths towards mechanistically dissecting trophoblast invasion in humans. Single-cell studies^15,16^ show that these models are promising but a comprehensive benchmarking has been lacking. 

Here we present a spatially resolved single-cell multiomic characterization of the maternal–fetal interface. We examine the site of placentation from historical samples of first trimester hysterectomies, which include the entire uterus containing the placenta, decidua and myometrium. Spatiotemporal ordering of trophoblast invasion enables us to predict the potential participants regulating placentation. We use this comprehensive detailed account of trophoblast differentiation to benchmark existing PTO and TSC models. Finally, we describe the interactions between trophoblast subsets and decidual cells that are likely to affect how arterial transformation by trophoblast occurs. Thus, we provide a description of the whole trajectory of human trophoblast cell states in the first trimester and their spatial niches.

## A spatial map of human placental bed

**Fig. 1:**
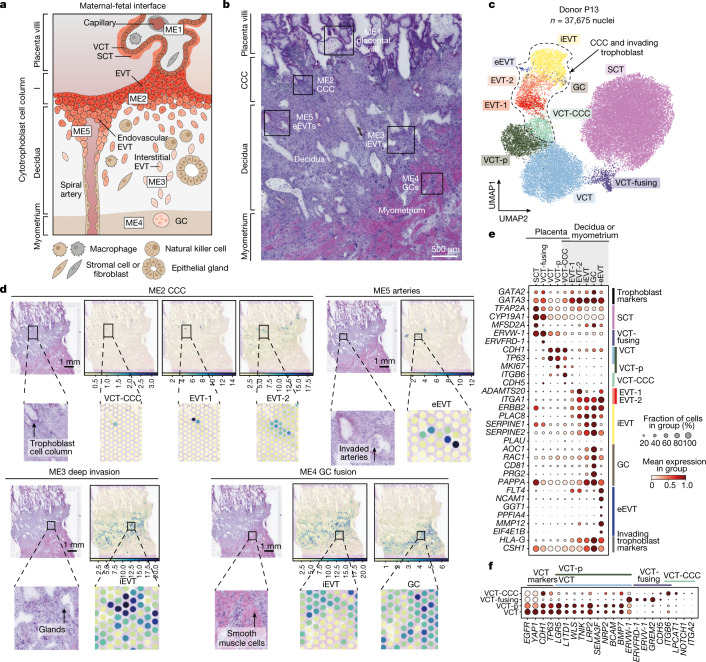
Trophoblast cell states in the early maternal–fetal interface. **a**, Schematic representation of the maternal–fetal interface during the first trimester of human pregnancy. **b**, Histological overview (haematoxylin and eosin (H&E) staining) of the implantation site of donor P13 (approximately 8–9 PCW) (*n* = 1). Black outlines indicate trophoblast microenvironments in space. **c**, Uniform manifold approximation and projection (UMAP) plot of snRNA-seq of donor P13 trophoblast nuclei in the maternal–fetal interface (*n* = 37,675 nuclei) coloured by cell state. **d**, Overview of spatial locations of invading trophoblast cell states in Visium spatial transcriptomics data of a section of donor P13 tissue (the position of the capture area is indicated with an arrow in Extended Data Fig. 1d). Spot colour indicates cell state density computed by cell2location, which is the number of cells of a given cell state in a Visium spot. Invading trophoblast cell states are grouped on the basis of the spatial microenvironment that they represent. **e**, Dot plots showing normalized, log-transformed and variance-scaled expression of genes (*y*-axis) characteristic of trophoblast cell states (*x*-axis) in donor P13 snRNA-seq data. **f**, Dot plots showing normalized, log-transformed and variance-scaled expression of genes (*x*-axis) characteristic of villous cytotrophoblast (*y*-axis) in donor P13 snRNA-seq data.

**Extended Data Fig. 1:**
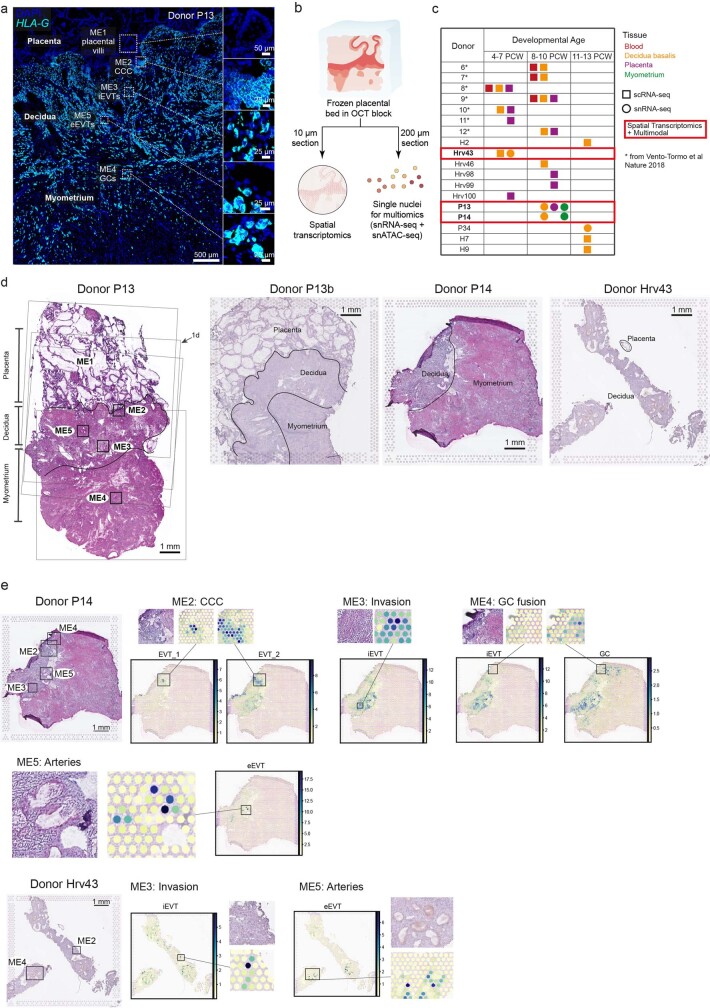
Spatial transcriptomics of human placental bed. **a**: High-resolution imaging of a section of the placenta-decidua interface stained by in situ hybridization (smFISH) for *HLA-G*, illustrating the depth of invasion of EVTs into the uterus (n = 1). Magnified insets (dashed squares) highlight the HLA-G-negative placental villi, and *HLA-G*+ EVTs emerging from the CCC to invade the decidua and myometrium. **b**: Overview of experimental design of the study. **c**: Cohort composition split by gestational age window (post-conceptional weeks, PCW) representing tissues sampled from each donor and performed assays. Highlighted in red rectangles are the three donors whose tissues have been additionally profiled with spatial transcriptomics (Visium) and multiome assays. **d**: Histological overview (H&E staining) of donors P13, P14 and Hrv43 tissues with annotations of tissue regions. For the implantation site of donor P13 (~ 8-9 PCW, left); black squares (small) indicate trophoblast microenvironments in space; faint grey squares (large) indicate positioning of tissue on Visium spatial transcriptomics capture areas; arrow indicates representative Visium section further explored in Fig. 1d. For Visium, P13 (n = 5 feature areas, 4 consecutive slides with overlapping positions and 1 slide from an additional tissue block - P13b), P14 (n = 2 feature areas, consecutive slides with same position), Hrv43 (n = 1 feature area). **e**: Cell state locations (derived with cell2location) in representative Visium sections of donors P14 and Hrv43 highlighting relevant spatial trophoblast microenvironments. Spot colour indicates cell state densities computed by cell2location as the number of cells of a given cell state in a Visium spot. Cytotrophoblast cell column (CCC), extravillous trophoblast (EVT), interstitial EVT (iEVT), giant cells (GC), endovascular EVT (eEVT), single-cell RNA sequencing (scRNA-seq), single-nuclei RNA sequencing (snRNA-seq), microenvironment (ME), Hematoxylin and Eosin (H&E).

**Extended Data Fig. 2:**
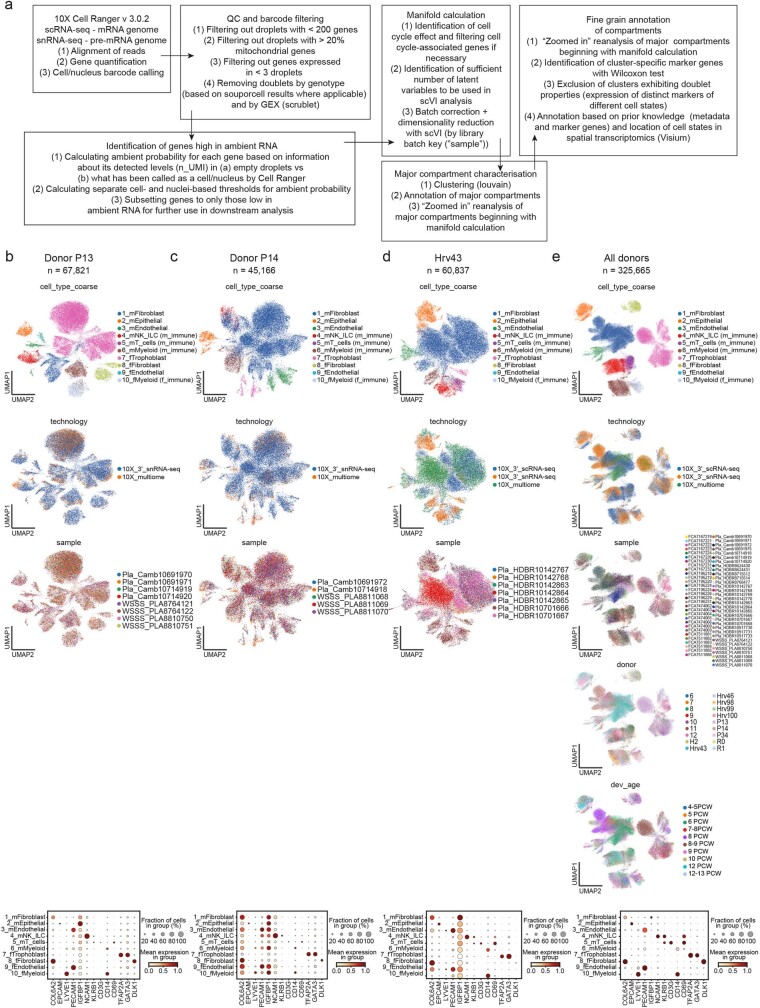
scRNA-seq and snRNA-seq data quality control and data analysis overview. **a**: Overview of the computational pipeline implemented for analysis of *in vivo* scRNA-seq and snRNA-seq data. Data integrated with scVI. **b**–**e**: (top) UMAP (uniform manifold approximation and projection) scatterplots of donors P13 (n = 67,821 nuclei), P14 (n = 45,166 nuclei), Hrv43 (n = 60,837 nuclei and cells) and all donors’ (n = 325,665 nuclei and cells, m = 18 donors) scRNA-seq and snRNA-seq data (b-e respectively) for all recovered cell states, coloured by coarse grain compartment annotation and metadata labels: assay, sample (10X library), donor and developmental age. (bottom) Dot plots show normalised, log-transformed and variance-scaled expression of genes characteristic of coarse grain compartment (X-axis) in donors profiled (Y-axis). Single-cell RNA sequencing (scRNA-seq), single-nuclei RNA sequencing (snRNA-seq), maternal (m), fetal (f), natural killer (NK), innate lymphocytes (ILC), single-cell RNA sequencing (scRNA-seq), single-nuclei RNA sequencing (snRNA-seq).

We profiled three human implantation sites (between 6 and 9 post-conceptional weeks (PCW)) using a multimodal approach (Fig. 1a,b, Extended Data Fig. 1a,b and Supplementary Tables 1–3). Consecutive sections from frozen tissue blocks of the implantation site were used for: (1) single-nuclei RNA sequencing (snRNA-seq); (2) combined snRNA-seq and single-nuclei assay for transposase-accessible chromatin with sequencing (snATAC–seq) (we refer to the combined analysis as the multiome); and (3) spatial transcriptomics using Visium (Extended Data Figs. 1c and 2a–d). To account for the large tissue area of one donor (P13), we targeted four consecutive sections with four spatial transcriptomics capture areas (Extended Data Fig. 1d). We also profiled five decidual and three placental samples from 5–13 PCW by scRNA-seq and snRNA-seq and integrated all the data with our previous scRNA-seq dataset of the maternal–fetal interface^13^ (Extended Data Figs. 1c and 2e). Our single-cell and spatial transcriptomics map is available at https://reproductivecellatlas.org.

**Extended Data Fig. 3:**
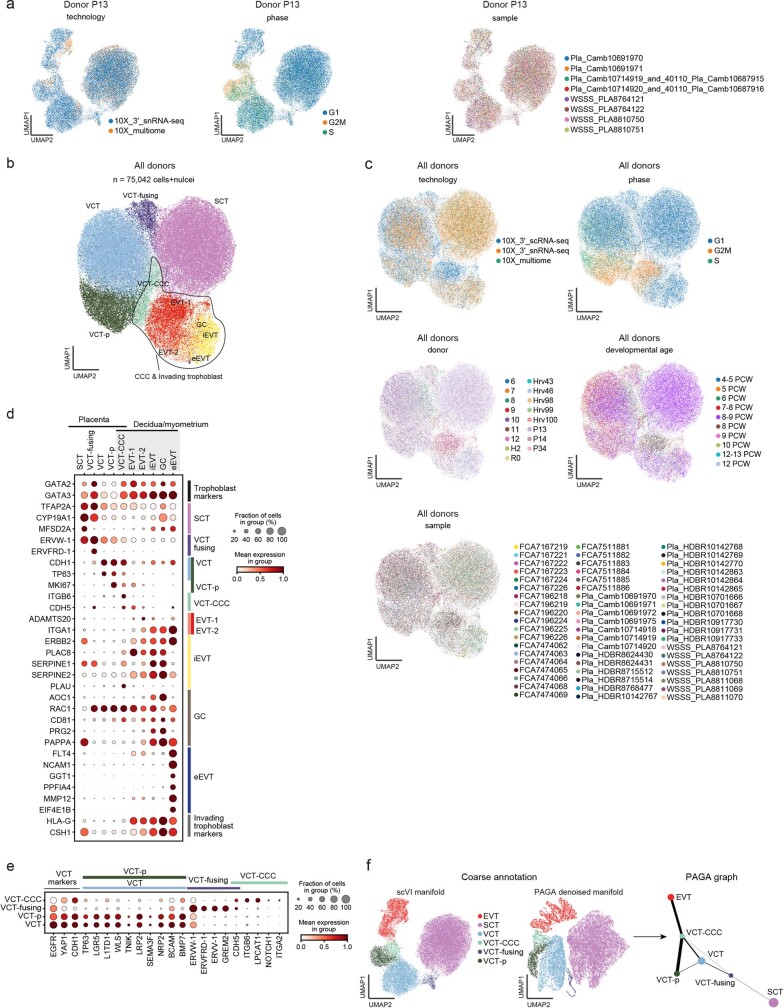
snRNA-seq and scRNA-seq trophoblast data analysis overview. **a**: UMAP (uniform manifold approximation and projection) scatterplots of donor P13 snRNA-seq data (n = 37,675 nuclei) for all trophoblast cell states coloured by (from left to right) assay, cell cycle phase of the nuclei and sample (10X library). Please note: bioinformatics analyses used cannot distinguish between G0 and G1. **b**: UMAP scatterplot of integrated snRNA-seq and scRNA-seq of all donors’ (n = 75,042 nuclei and cells, m = 17 donors with trophoblast present) trophoblast cell states in the maternal-fetal interface coloured by cell state. **c**: UMAP scatterplots of all donors’ scRNA-seq and snRNA-seq data for all donors’ (n = 75,042 nuclei and cells, m = 17 donors with trophoblast present) trophoblast cell states coloured by assay, sample (10X library), cell cycle phase of the cells/nuclei, donor and developmental age. **d**: Dot plot showing normalised, log-transformed and variance-scaled expression of genes (Y-axis) characteristic of trophoblast cell states (X-axis) in all donors (m = 17 donors with trophoblast present). **e**: Dot plot showing normalised, log-transformed and variance-scaled expression of genes (X-axis) characteristic of VCT cell states (Y-axis) in all donors (m = 17 donors with trophoblast present). **f**: Results of PAGA trajectory inference of all trophoblast cell states in donor P13 snRNA-seq data. (left) main manifold, center: denoised PAGA manifold, (right) PAGA reconstruction of putative trajectory tree for all trophoblast cell states. For the purpose of this analysis all EVTs have been united in annotation under the ‘EVT’ label. Syncytiotrophoblast (SCT), villous cytotrophoblast (VCT), cytotrophoblast cell column (CCC), proliferative (p), extravillous trophoblast (EVT), interstitial EVTs (iEVTs), giant cells (GC), endovascular EVT (eEVT), single-cell RNA sequencing (scRNA-seq), single-nuclei RNA sequencing (snRNA-seq).

We examined trophoblast heterogeneity in two steps. First, we analysed the full-thickness implantation site from P13 (at around 9 PCW), as it contains both fetal (placenta) and maternal (decidua and myometrium) tissues on the same slide, and the tissue block is perfectly preserved and oriented (Fig. 1c and Extended Data Fig. 3a). Second, we validated the trophoblast populations and their markers in the integrated dataset (around 5–13 PCW) (Extended Data Fig. 3b,c). Trophoblast subsets were annotated by considering canonical markers and their spatial location (Fig. 1d–f and Extended Data Figs. 1e and 3d,e). To assign spatial coordinates we used cell2location^17^, our probabilistic method to deconvolve the spatial voxels using our pre-defined snRNA-seq data. We then placed the trophoblast cells into five pre-defined microenvironments (ME1–ME5) in the tissue based on manual histological annotation.

In the placental villi (ME1), villous cytotrophoblast (VCT) fuse to form the overlying SCT layer that is in contact with maternal blood in the intervillous space. VCT subsets express high levels of *TP63* and *CDH1* in the P13 donor (Fig. 1e) and all other donors (Extended Data Fig. 3d). VCT and VCT-proliferative (VCT-p) upregulate known stem and progenitor cell markers (*LGR5*, *L1TD1* and *TP63*), Wnt signalling molecules (*WLS* and *TNIK*), the SEMA3F–NRP2 signalling complex and the VCT marker *BCAM*^18^ (Fig. 1f, Extended Data Fig. 3e). We define an additional population of VCT in the placental villi that we name VCT-fusing, which the connectivity network PAGA^19^ indicates is an intermediate cell state between VCT and SCT (Extended Data Fig. 3f). As VCT commit into VCT-fusing, they downregulate Wnt (*WLS*, *TNIK* and *LGR5*) and BMP signals (*BMP7* and upregulation of BMP antagonist *GREM2*), and upregulate the endogenous retroviral genes (*ERVW-1, ERVFRD-1, ERVV-1*) known to mediate trophoblast fusion^20^ (Fig. 1f and Extended Data Fig. 3e). Our strategy for isolation of nuclei enables the capture of mature multinucleated SCTs (expressing *CYP19A1* and *MFSD2A*), which were not found in previous scRNA-seq studies^13,21^ (Fig. 1e and Extended Data Fig. 3d).

Soon after implantation, foci of cytotrophoblast cell columns (CCCs) arise from the VCTs that break through the SCT. These expand and form a shell around the conceptus that becomes discontinuous in the following weeks. EVTs begin to differentiate in cell columns but invasive EVTs emerge only when the anchoring villi attach to the maternal decidua. In the trophoblast shell (ME2), we define an additional population of CCC VCT (VCT-CCC) (Fig. 1d and Extended Data Fig. 1e). VCT-CCCs are proliferative and PAGA analysis shows they are likely to emerge from VCT or VCT-p and give rise to EVTs (Extended Data Fig. 3f). This analysis confirms that VCT is a common progenitor for both VCT-fusing, giving rise to SCT, and VCT-CCC where EVTs emerge. As they commit to become VCT-CCCs, they downregulate the Wnt pathway (*WLS*, *TNIK* and *LGR5* expression), upregulate *NOTCH1*, undergo an integrin shift (upregulating *ITGB6* and *ITGA2*), and upregulate markers characteristic of epithelial–mesenchymal transition^22^ (*LPCAT1*) (Fig. 1f and Extended Data Fig. 3e). Expression of *NOTCH1* and *ITGA2* is characteristic of putative trophoblast progenitor cells located in a small niche in the CCC^23,24^. In agreement with this finding, in ME2, VCT-CCCs co-localize with EVTs (Fig. 1d and Extended Data Fig. 1e).

## Trajectories of EVT defined by StOrder

**Fig. 2:**
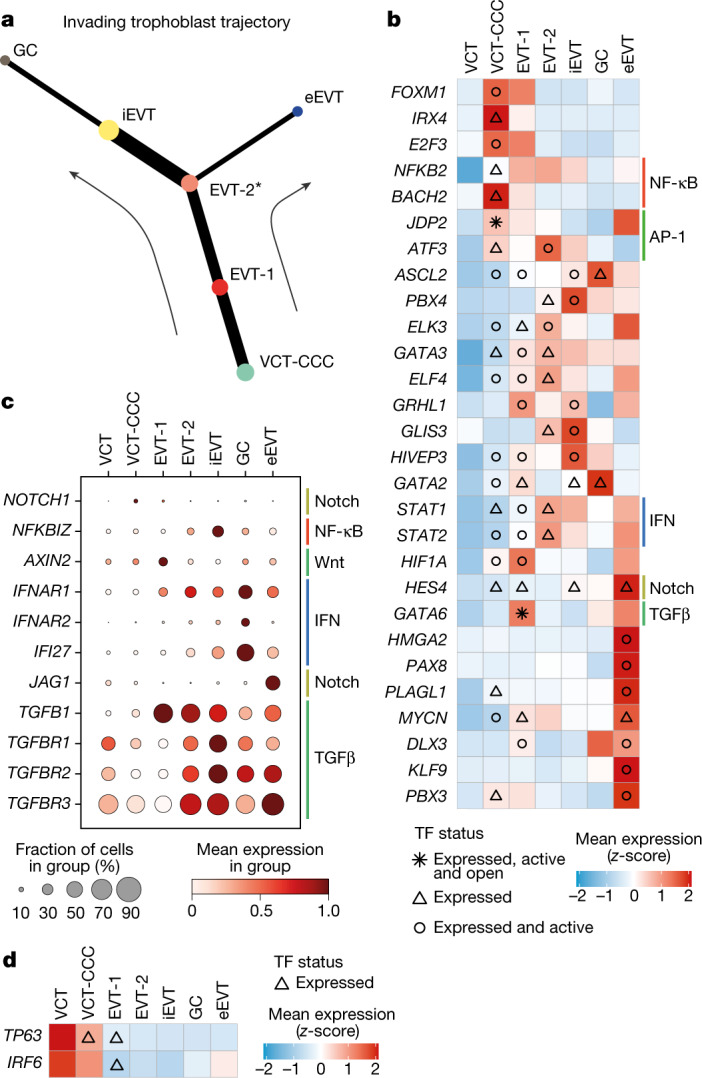
Transcription factors that are active during EVT invasion. **a**, Representative tree of EVT differentiation trajectory inferred by StOrder (Methods). The tree shown is inferred with *α* = 0.4 and *β* = 0.5 for snRNA-seq and spatial transcriptomics data from donors P13 (5 capture areas), P14 (2 capture areas) and Hrv43 (1 capture area). Tree edge thickness is proportional to connectivity (joint measure inferred from snRNA-seq data and spatial transcriptomics data) between two cell types connected by that edge. The asterisk indicates the bifurcation point. **b**, Heat map showing *z*-scores of normalized, log-transformed and scaled expression of transcription factor (TF) genes upregulated during trophoblast invasion in donor P13 snRNA-seq data. The *x*-axis indicates cell state, the *y*-axis lists transcription factors. Differential expression (upregulated genes) is tested along the invading trophoblast trajectory (as shown in **a**) in a retrograde manner using the limma approach (false discovery rate (FDR) < 0.05, with Bonferroni correction for multiple hypotheses testing). Coloured bars to the right of heat map indicate members of selected pathways. IFN, interferon. **c**, Dot plot showing normalized, log-transformed and variance-scaled expression of genes (*x*-axis) of signalling molecules upregulated in EVT (*y*-axis) in donor P13 snRNA-seq data. **d**, Heat map showing *z*-score of normalized, log-transformed and variance-scaled expression of transcription factors (*x*-axis) downregulated during trophoblast invasion in P13 in trophoblast states (*y*-axis). Differential expression (downregulated genes) is tested along invading trophoblast trajectory (as shown in **a**) in a retrograde manner using the limma approach (FDR < 0.05, with Bonferroni correction for multiple hypotheses testing).

**Extended Data Fig. 4:**
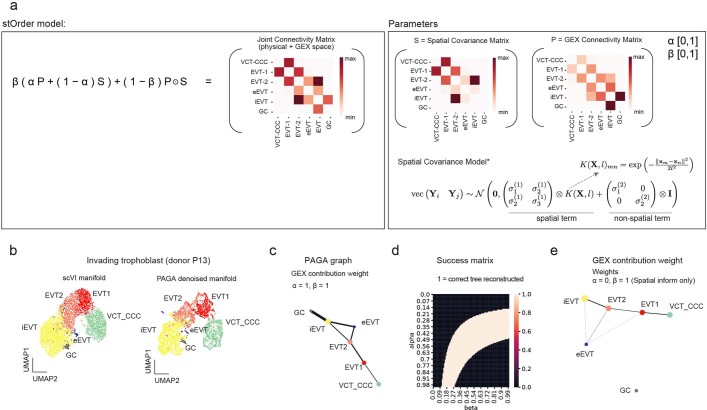
Analysis of extravillous trophoblast invasion trajectory using stOrder. **a**: Schematic overview of StOrder approach representing the workflow of joint cell differentiation trajectory inference from gene expression and spatial transcriptomics data (See methods). **b**: (Left) Main UMAP (uniform manifold approximation and projection) scatterplot and (right) denoised manifold used for PAGA trajectory inference of all trophoblast cell states in donor P13 single-nuclei RNA sequencing (snRNA-seq) data. **c**: PAGA reconstruction of putative trajectory tree for all extravillous trophoblast cell states. This corresponds to the trajectory tree inferred by stOrser with α = 1, β = 1 parameters from donor P13 snRNA-seq data and spatial transcriptomics data of donors P13 (5 capture areas), P14 (2 capture areas) and Hrv43 (1 capture area). **d**: Heatmap of binary success matrix of stOrder approach for pairs of (α,β), values (along Y and X axes, respectively). Assigned matrix value is 1 if a tree of correct topology has been reconstructed for that pair of (α,β) values, and 0 if no tree of correct topology was reconstructed. **e**: Reconstruction of putative invading trophoblast trajectory tree based solely on spatial data inferred by stOrder with α = 0, β = 1 parameters from donor P13 snRNA-seq data and spatial transcriptomics data of donors P13 (5 chips), P14 (2 chips) and Hrv43 (1 chip). Villous cytotrophoblast (VCT), cytotrophoblast cell column (CCC), extravillous trophoblast (EVT), interstitial EVTs (iEVTs), giant cells (GC), endovascular EVT (eEVT).

To further investigate the EVT differentiation pathway as it arises from the CCCs of the anchoring villi to infiltrate maternal tissue, we leveraged both spatial and single-cell transcriptomics data using a three-step statistical framework, which we named StOrder (Extended Data Fig. 4a and Methods). First, StOrder builds a gene expression-based connectivity matrix (generated in our case by PAGA^19^) to establish putative connections between clusters (Extended Data Fig. 4b). The values in this matrix are interpreted as pairwise similarity scores for cell states in the gene expression space. Second, StOrder generates a spatial covariance matrix that reflects the pairwise proximity of trophoblast states that co-exist in space. To do so, StOrder takes as an input the estimated cell densities per spot (derived in our case with cell2location^17^) in Visium spatial transcriptomics data, and fits a Gaussian process model that derives pairwise spatial covariance scores for all the cell state pairs (Extended Data Fig. 4a). This enables inference of which cell states are proximal in physical space and are probably gradually differentiating as they migrate. Third, StOrder reconstructs connections between cell states by combining the connectivity matrix (step 1) from single-cell transcriptomics data and the spatial covariance matrix (step 2) from the spatial data in a weighted manner (Fig. 2a and Extended Data Fig. 4a–e). In sum, StOrder reconstructs the likely cell transitions in space by taking into account both the single-cell transcriptomics and the mini-bulk spatial transcriptomics data.

**Extended Data Fig. 5:**
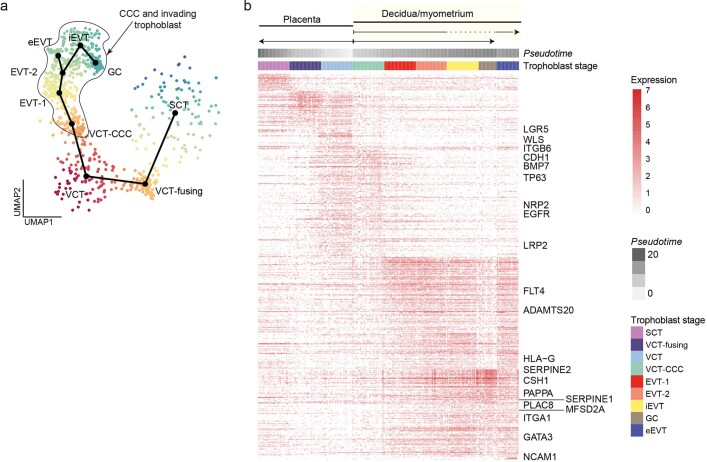
Analysis of extravillous trophoblast invasion trajectory using Slingshot. **a**: Minimum spanning tree of donor P13 trophoblast single-nuclei RNA sequencing (snRNA-seq) data computed by Slingshot, visualised on the UMAP (uniform manifold approximation and projection) embedding of P13 donor trophoblast cells from Fig. 1c (n = 37,675 nuclei). Bigger black dots indicate trophoblast states. Smaller dots’ colour indicates pseudotime. **b**: Heatmap showing normalised and log-transformed expression values of the 567 genes associated with trophoblast pseudotime, estimated with tradeSeq (p-value < 1x10-6 and mean LogFC > 0.5). Cells in rows are sorted according to the predicted pseudotime. Genes are sorted according to the trophoblast state where gene expression peaks. Marker genes are highlighted. Syncytiotrophoblast (SCT), villous cytotrophoblast (VCT), cytotrophoblast cell column (CCC), extravillous trophoblast (EVT), interstitial EVTs (iEVTs), giant cells (GC), endovascular EVT (eEVT).

StOrder enabled us to resolve the most likely trajectory for the emergence and differentiation of invasive EVTs (Fig. 2a). Consistent trajectories were obtained when reconstructing pseudotime on snRNA-seq data using Slingshot^25^ (Extended Data Fig. 5a). We then calculated differentially expressed genes (DEGs) along the three trophoblast trajectories with different end points: (1) eEVT, (2) GC and (3) SCT (Extended Data Fig. 5b and Supplementary Table 5). VCT-CCCs are the precursors of EVTs-1 and EVTs-2 and co-localize with them in ME2 (Fig. 1d and Extended Data Fig. 1e). EVTs-1 are proliferative while EVTs-2 do not proliferate and upregulate the metalloprotease gene *ADAMTS20* and the integrin subunit gene *ITGA1* (Fig. 1e and Extended Data Fig. 3d). EVTs-2 are located at the distal end of the anchoring villi, and are identified as the bifurcation point between eEVTs and interstitial EVTs (iEVTs) (Figs. 1d and 2a). Thus, EVTs-2 can transition either into iEVTs that invade through decidual stroma, or into eEVTs that move down inside the arteries.

**Extended Data Fig. 6:**
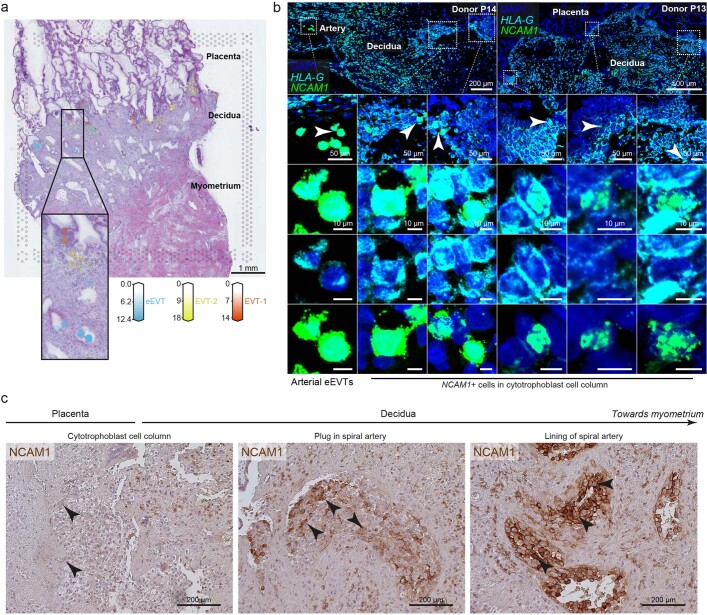
Histological characterisation of eEVTs. **a**: Estimated amount of mRNA computed by cell2location (colour intensity) contributed by each cell population to each spot (colour) shown over the hematoxylin and eosin (H&E) image of donor P13 implantation site (n = 1). **b**: (Top) High-resolution imaging of sections of the placenta-decidua interface from two donors (n = 2, donor ID is indicated in each panel), stained by multiplexed single molecule fluorescence in situ hybridization (smFISH) for *HLA-G* and *NCAM1*; dashed squares indicate areas shown magnified below. (Middle) magnified insets highlight an artery containing aggregating eEVTs (left) and cytotrophoblast cell columns; in the latter, solid arrows indicate sporadic nascent *NCAM1*+ cells shown magnified below (bottom). **c**: Expression of NCAM1 (marker of eEVT) with IHC in first-trimester decidual tissue. Nuclei are counterstained with hematoxylin. Representative images from three different donors (n = 3).

eEVTs are present inside spiral arteries (ME5) (Fig. 1d and Extended Data Fig. 1e). Besides *NCAM1*^26,27^, eEVTs also upregulate *GGT1*, *PPFIA4* and *MMP12* (Fig. 1e and Extended Data Fig. 3d). Evidence that eEVTs emerge from the distal end of the CCC is supported by their close proximity to EVTs-2 (Extended Data Fig. 6a). In our samples, we detect sporadic *NCAM1*^*+*^ cells close to the cytotrophoblast shell when it is overlying a spiral artery, by single-molecule fluorescent in situ hybridisation (smFISH) (Extended Data Fig. 6b). Immunohistochemistry confirms our previous findings^26,27^ that cells in the CCC do not stain with a monoclonal antibody to NCAM1, but there are scattered positive cells in the plug of eEVTs beneath this column. In a more proximal portion of the same artery all the eEVTs lining the artery are NCAM1^+^ (Extended Data Fig. 6c).

Highly invasive iEVTs are found in ME3, surrounded by decidual stromal and immune cells (Fig. 1d and Extended Data Fig. 1e). iEVTs upregulate *PLAC8*^28^ and plasminogen activator inhibitor genes *SERPINE1* and *SERPINE2*, with concomitant downregulation of the plasminogen activator gene *PLAU* (Fig. 1e and Extended Data Fig. 3d). iEVTs eventually fuse to form placental bed GCs deeper in the decidua and myometrium (ME4) (Fig. 1d and Extended Data Fig. 1e). GCs upregulate *RAC1* and *CD81*, and the PRG2–PAPPA complex^29^ (Fig. 1d,e, Extended Data Fig. 1e and Extended Data Fig. 3c).

**Extended Data Fig. 7:**
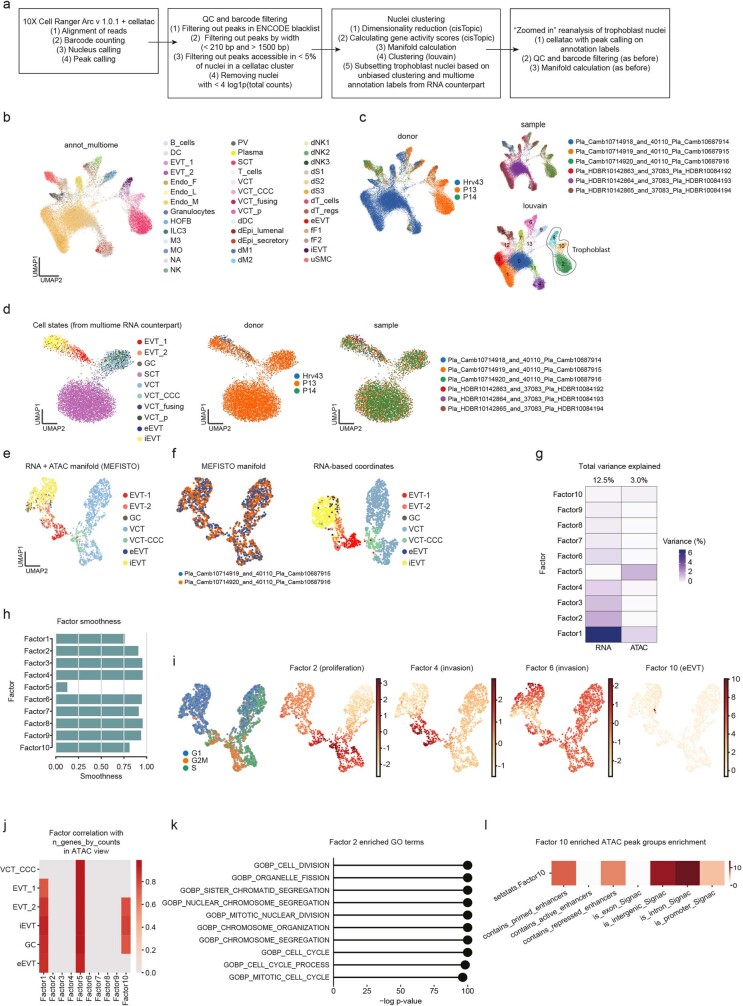
Multimodal analysis of extravillous trophoblast invasion. **a**: Overview of the computational pipeline implemented for analysis of multimodal data. **b**-**c**: UMAP (uniform manifold approximation and projection) scatterplot of multimodal snATAC-seq data from donors P13, P14 and Hrv43 (n = 52,798 nuclei) coloured by cell state (b), donor, sample (10X library) or unbiased clustering labels (c). Data is annotated based on the corresponding single-nuclei RNA sequencing (snRNA-seq) cell state assignment. **d**: UMAP scatterplot of integrated multimodal single-nuclei ATAC sequencing (snATAC-seq) data for trophoblast only from donors P13, P14 and Hrv43 (n = 7449 nuclei) coloured by cell state, donor and sample (10X library). **e**: UMAP scatterplot of multiome (snRNA-snATACseq) data of invading trophoblast cells from donor P13 (n = 1605 nuclei) coloured by cell state. The manifold is calculated based on dimensionality reduction performed by MEFISTO (model with n = 9 factors). **f**: (Left) UMAP scatterplot of multiome (snRNA-snATACseq) data of invading trophoblast cells from donor P13 (n = 1605 nuclei) coloured by sample. The manifold is calculated based on dimensionality reduction performed by MEFISTO (model with n = 9 factors). (Right) Scatterplot of UMAP coordinates obtained from the RNA expression data that were used as covariates for MEFISTO, coloured by cell state. **g**: Heatmap representing percentage of variance explained by each MEFISTO factor in each data modality. **h**: Smoothness along differentiation estimated with MEFISTO. **i**: UMAP scatterplot of multiome (snRNA-snATACseq) data of invading trophoblast cells from donor P13 (n = 1605 nuclei) coloured by cell cycle phase and MEFISTO factor values for important selected factors. **j**: Spearman’s rank correlation coefficients of each latent factor learned with MEFISTO and the number of genes per counts in snATAC-seq data (multiome). **k**: Gene set (RNA) enrichment analysis overview of MEFISTO factor 2 using two-sided parametric t-test, FDR is used to rank gene sets. **l**: Peak set (ATAC) enrichment analysis overview of MEFISTO factor 10 using two-sided parametric t-test, FDR is used to rank peak sets. Villous cytotrophoblast (VCT), cytotrophoblast cell column (CCC), proliferative (p), extravillous trophoblast (EVT), interstitial EVTs (iEVTs), giant cells (GC), endovascular EVT (eEVT), dendritic cells (DC), lymphatic (l), maternal (m), fetal (f) Hofbauer cells (HOFB), innate lymphocytes (ILC), macrophages (M), monocytes (MO), natural killer (NK), perivascular (PV), decidual (d), epithelial (epi), stromal (S), fibroblasts (F), uterine smooth muscle cells (uSMC).

We next explored the regulatory programmes that might mediate EVT invasion by analysing the multimodal RNA-seq and ATAC–seq data (Extended Data Fig. 7a–c). We applied our multifactorial method MEFISTO^30^ to donor P13 multimodal data, which contained the full spectra of VCT and EVT subsets (Extended Data Fig. 7d–f). MEFISTO identified 10 latent factors that jointly explain 12.5% of the variance in the RNA expression data and 3% of the chromatin accessibility data (Extended Data Fig. 7g,h). Using a logistic regression approach, we define factors 2, 4, 6 and 10 as the main driving factors of the trophoblast trajectory (Extended Data Fig. 7i–l). Factors 2, 4 and 6 explain changes along the main interstitial trophoblast invasion pathway (VCT-CCC to GC) (Supplementary Table 4). Genes contributing strongly to these factors are *MKI67*, *CENPK* (cell cycle, factor 2); *CSF1R*, *ADAM8* and *LAIR2* (early trophoblast invasion, factor 4); *CALD1* and *COL21A1* (late trophoblast invasion, factor 6). Factor 10 captured eEVTs; the main genes contributing to this factor include *NCAM1*, *JAG1*, *ADORA1*, *EPHA1* and *HES4*.

## Transcription factors in EVT subsets

**Extended Data Fig. 8:**
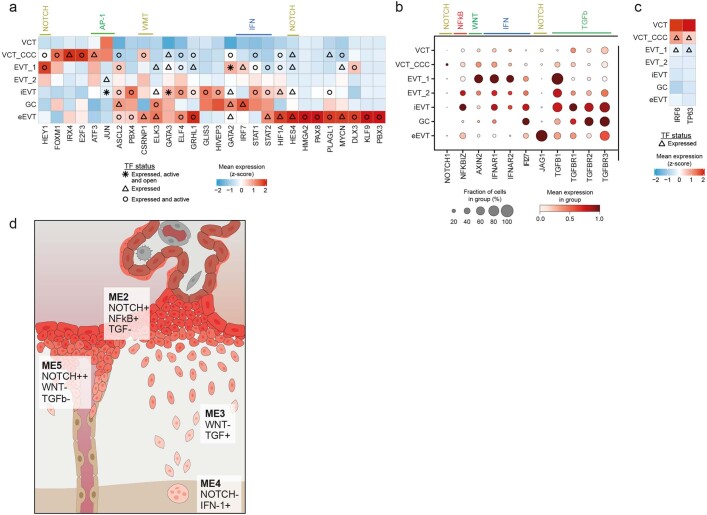
Transcription factors active in extravillous trophoblast cell states (all donors). **a**: Heatmap showing z-score of normalised, log-transformed and variance-scaled expression of transcription factors (TFs) upregulated during trophoblast invasion in all donors (n = 17 donors with trophoblast present). Y-axis indicates cell state, X-axis lists TFs. Differential expression (upregulated genes) is tested along invading trophoblast trajectory (as shown in Fig. 2a) in a retrograde manner using limma approach (FDR < 0.05, with Bonferroni correction for multiple hypotheses testing. **b**: Dot plot showing normalised, log-transformed and variance-scaled expression of genes (X-axis) of signalling molecules upregulated in EVT (Y-axis) in all donors (n = 17 donors with trophoblast present). **c**: Heatmap showing z-score of normalised, log-transformed and scaled expression of TFs downregulated during trophoblast invasion in all donors (n = 17 donors with trophoblast present). Y-axis indicates cell state, X-axis lists TFs. Differential expression (downregulated genes) is tested along invading trophoblast trajectory (as shown in Fig. 2a) in a retrograde manner using limma approach (FDR < 0.05, with Bonferroni correction for multiple hypotheses testing). **d**: Schematic representation of signalling pathways in distinct spatial microenvironments. Villous cytotrophoblast (VCT), cytotrophoblast cell column (CCC), extravillous trophoblast (EVT), interstitial EVTs (iEVTs), giant cells (GC), endovascular EVT (eEVT), microenvironment (ME), transcription factors (TFs).

To identify the major regulatory programmes driving EVT differentiation, we extracted the transcription factors that are differentially expressed and active along the EVT differentiation trajectory (Supplementary Table 6 and Methods). Activation of the *FOXM1*–*NOTCH1* axis is likely to lead to the differentiation of VCTs into VCT-CCCs (Fig. 2b,c and Extended Data Fig. 8a,b). Upregulation of *NOTCH1* may trigger the downregulation of *IRF6* and *TP63* expression in trophoblast^23,31^ (Fig. 2d and Extended Data Fig. 8c). VCT-CCCs upregulate NF-κB pathway genes (*NFKB2* and *BACH2*) and modulate AP-1 signalling genes (*JDP2* and *ATF3*), which may result in epithelial–mesenchymal transition (Fig. 2b and Extended Data Fig. 8a). Activation of the NF-κB pathway is maintained throughout EVT differentiation (Fig. 2b and Extended Data Fig. 8a), but there is upregulation of the NF-κB inhibitor (*NFKBIZ*) at the iEVT stage (Fig. 2c and Extended Data Fig. 8b). This could be another mechanism to avoid inflammation as EVTs invade^13,32^.

Invading EVTs intermingle with stromal and immune cells in the decidua. Decidual stromal cells secrete the Wnt inhibitor DKK1^33^ and EVT invasion is characterized by inhibition of Wnt, with downregulation of the Wnt target *AXIN2* (Fig. 2c and Extended Data Fig. 8b). As they invade, iEVTs upregulate the transcription factor ASCL2^34^, other transcription factors involved in cancer invasion (*ELK3–GATA3* complex^35^), as well as tumour suppressor genes (*GRHL1*) (Fig. 2b and Extended Data Fig. 8a). This is in keeping with iEVTs being non-proliferative. As iEVTs transition into GCs, they upregulate receptors of the type I interferon pathway (*IFNAR1* and *IFNAR2*) and its targets (*IFI27*) (Fig. 2c and Extended Data Fig. 8b).

The eEVTs interact with endothelial cells, which they replace, and constituents of maternal blood. eEVTs have a unique pattern of transcription factor genes, which include *HMGA2*, *PAX8*, *PLAGL1*, *MYCN* and *PBX3* (Fig. 2b and Extended Data Fig. 8a). In addition, eEVTs upregulate Notch signalling (*HES4* and *JAG1*) and the expression of TGFβ signalling genes (*TGFB1*, *TGFBR1* and *TGFBR2*) is lower than in iEVT (Fig. 2c,d and Extended Data Fig. 8a,b). *GATA6*, which is known to affect vessels by suppressing autocrine TGFβ signalling^36^, is always upregulated and active in EVT-1 and maintains its high expression in eEVTs, as opposed to iEVTs. In summary, eEVT identity is marked by strong upregulation of Notch signalling and downregulation of TGFβ signalling, whereas iEVT cell fate is characterized by TGFβ upregulation and Wnt inhibition (Extended Data Fig. 8d).

## Benchmarking of trophoblasts in vitro

**Fig. 3:**
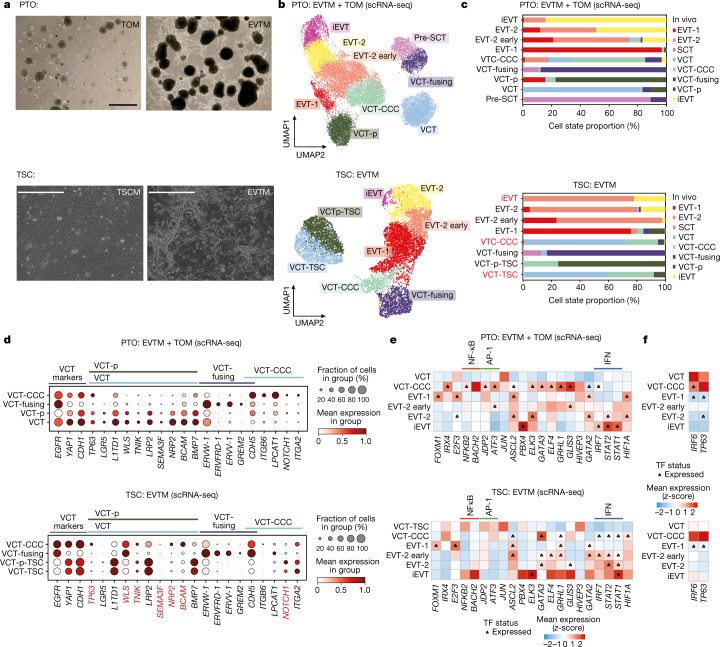
Benchmark of EVTs derived from primary-derived trophoblast organoids and TSCs. **a**, Top, phase-contrast images of PTOs plated in a Matrigel droplet and exposed to TOM or EVTM. Scale bar, 1 mm. Representative image of *n* = 6 experiments. Below, phase-contrast images of TSCs exposed to TSCM or EVTM. Scale bar, 400 μm. Representative image of *n* = 2 experiments. **b**, UMAP plot of PTO (*n* = 26,852 cells) and TSC (n = 9957 cells) scRNA-seq data coloured by cell state. Annotation was performed as indicated in Extended Data Fig. 9b. **c**, Bar plot representing the proportion (%) of cell states assigned to the in vitro cell states (defined by markers) using a logistic regression classifier trained on the in vivo data. Red text indicates cell states that differ between the annotations given by the logistic regression classifier and the ones given by the expression of canonical markers. **d**, Dot plot showing normalized, log-transformed and variance-scaled expression of genes (*x*-axis) characteristic of VCT *y*-axis in PTOs (top) and TSCs (bottom). Red text indicates genes that differ from the in vivo observed expression pattern. **e**, Heat map showing *z*-scores of normalized, log-transformed and variance-scaled expression of transcription factor genes that are known to be upregulated in in vivo trophoblast invasion (see Fig. 2b). The *y*-axis indicates cell state and the *x*-axis shows transcription factor genes. **f**, Heat map showing *z*-scores of normalized, log-transformed and variance-scaled expression of transcription factor genes that are known to be downregulated in in vivo trophoblast invasion.

**Extended Data Fig. 9:**
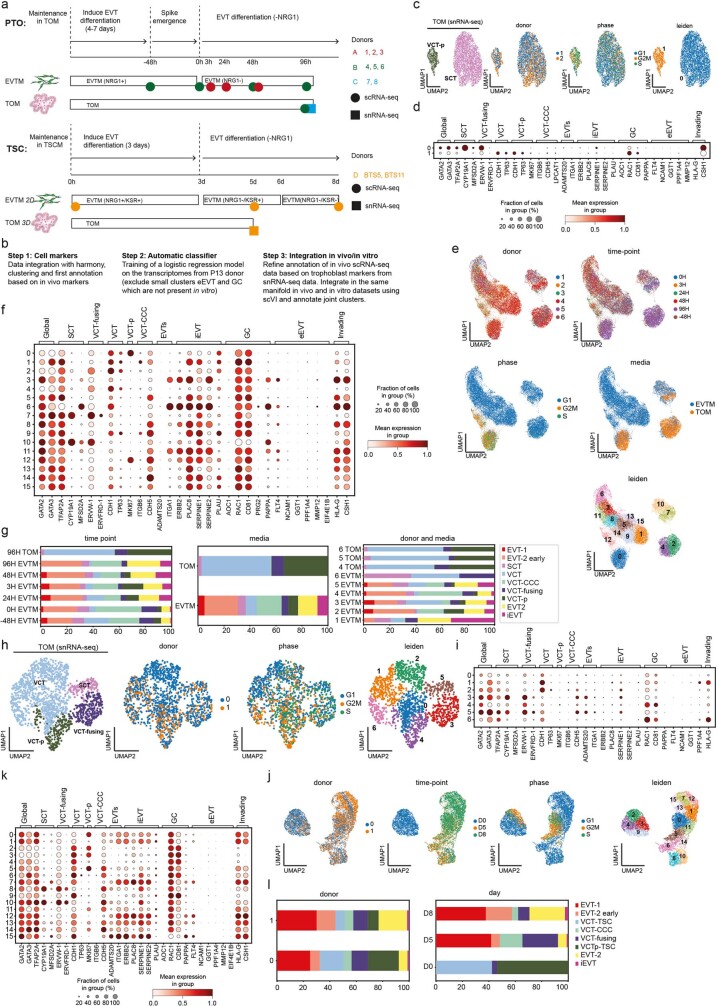
scRNA-seq and snRNA-seq data quality control and analysis overview of the trophoblast *in vitro* models. **a**: Schematic representation of the extravillous trophoblast (EVT) differentiation experimental design, indicating time points and biological replicates in both primary trophoblast organoids (PTO, n = 6 scRNA-seq and n = 2 for snRNA-seq) and trophoblast stem cell (TSC, n = 2 for both scRNA-seq and snRNA-seq) models. **b**: Diagram showing the annotation of the *in vitro* models. Firstly, we analysed the datasets in four separate manifolds and annotated each of the cell states based on canonical markers. Secondly, we projected the trophoblast *in vivo* reference data onto the *in vitro* trophoblast subsets by building a logistic regression classifier that we trained on P13 snRNA-seq in vivo dataset. We excluded eEVT and GC cells as these are scarcely represented and our marker data show they are not present in the *in vitro* cultures. Thirdly, we integrated scRNA-seq data from *in vivo* and *in vitro* conditions using scVI, and used this manifold to calculate differentially expressed genes (DEG) amongst subsets. **c**: UMAP (uniform manifold approximation and projection) scatterplots of snRNA-seq (n = 3928 nuclei) of PTOs coloured by cell state, donor (n = 2 donors), cell cycle phase and unbiased clustering using leiden. Sample integration was performed with Harmony. **d**: Dot plot showing normalised, log-transformed and variance-scaled expression of genes (X-axis) of main trophoblast subsets (Y-axis) in each of the clusters identified by unbiased clustering (a) in snRNA-seq of primary trophoblast organoids (PTOs). **e**: UMAP scatterplots of scRNA-seq of PTOs derived from n = 6 donors and coloured by donor, time-point, cell cycle phase, media cultured and unbiased clustering using leiden. **f**: Dot plot showing normalised, log-transformed and variance-scaled expression of genes (X-axis) of main trophoblast subsets in each of the clusters identified by unbiased clustering (c) in scRNA-seq of PTOs (Y-axis). **g**: Bar plots showing the proportion (%) of final cell states identified in data of each time point (left), media (center) and donor (right) for PTOs scRNA-seq. **h**: UMAP scatterplots of snRNA-seq (n = 1563 nuclei) of trophoblast organoids from trophoblast stem cells (TSC) coloured by cell state, donor, cell cycle phase and unbiased clustering using leiden. Sample integration was performed with Harmony. **i**: Dot plot showing normalised, log-transformed and variance-scaled expression of genes (X-axis) of main trophoblast subsets (Y-axis) in each of the clusters identified by unbiased clustering (f) in snRNA-seq of trophoblast stem cells (TSC). **j**: UMAP scatterplots of scRNA-seq of EVT derived from TSC coloured by donor, time-point, cell cycle phase and unbiased clustering using leiden. **k**: Dot plot showing normalised, log-transformed and variance-scaled expression of genes (X-axis) of main trophoblast subsets in each of the clusters identified by unbiased clustering (j) in scRNA-seq of trophoblast stem cells (TSC) (Y-axis). **l**: Bar plots showing the proportion (%) of final cell states identified in each donor’s data (left) and time-point (right) for TSCs scRNA-seq. Trophoblast organoid media (TOM), syncytiotrophoblast (SCT), villous cytotrophoblast (VCT), cytotrophoblast cell column (CCC), proliferative (p), extravillous trophoblast (EVT), EVT media (EVTM), interstitial EVT (iEVT), giant cells (GC), endovascular EVT (eEVT), single-cell RNA sequencing (scRNA-seq), single-nuclei RNA sequencing (snRNA-seq).

**Extended Data Fig. 10:**
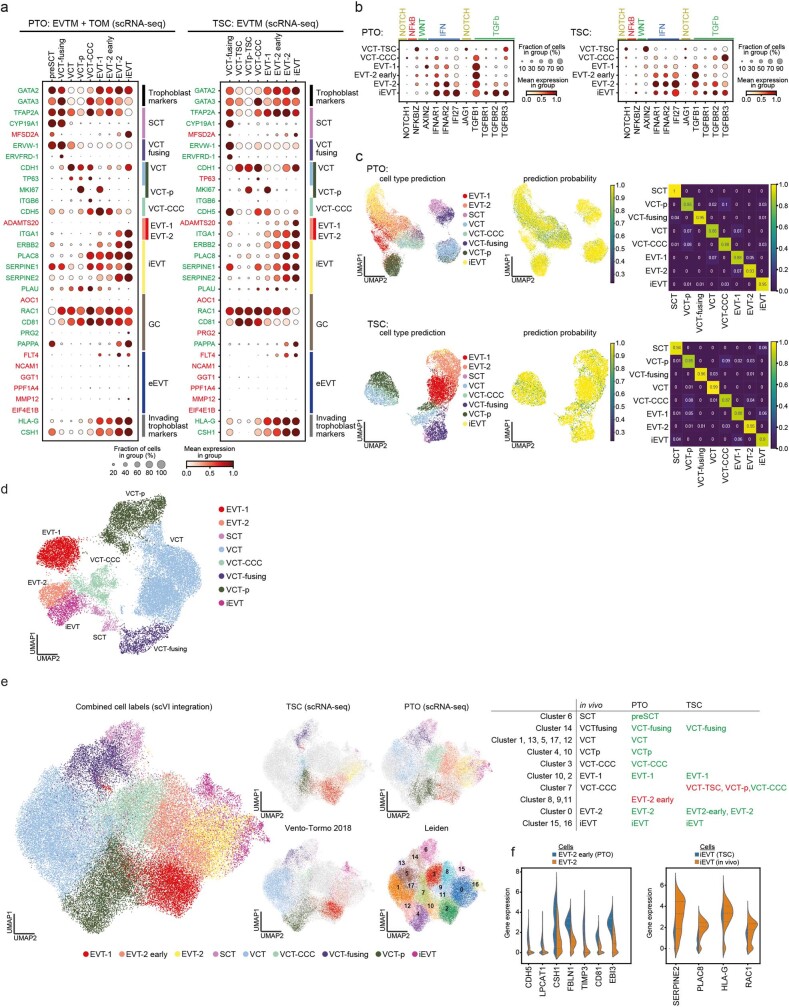
Annotation and benchmark of trophoblast *in vitro* models. **a**: Dot plots showing normalised, log-transformed and variance-scaled expression of genes (Y-axis) characteristic of trophoblast cell states (X-axis) in primary trophoblast organoids (PTO, left) and trophoblast stem cell (TSC, right) models. Marked in red are genes that differ from the *in vivo* observed expression pattern. **b**: Dot plot showing normalised, log-transformed and variance-scaled expression of genes (X-axis) of signalling molecules known to be upregulated in *in vivo* trophoblast invasion plotted in trophoblast organoids (Y-axis). **c**: UMAP (uniform manifold approximation and projection) scatterplot of PTO single-cell RNA sequencing (scRNA-seq data, n = 26,853 cells) coloured by predicted cell state (top left) and probability (top center) according to our logistic regression model. Only *in vivo* data from donor P13 (snRNA-seq) was considered for the training. Confusion matrix with predictions on test set based on common features with PTO (Top right). UMAP scatterplot of TSC scRNA-seq data (n = 9957 cells) coloured by predicted cell state (bottom left) and probability (bottom center) according to our logistic regression model. Only in vivo data from donor P13 (snRNA-seq) was considered for the training. Confusion matrix with predictions on test set based on common features with TSC (Bottom right). **d**: UMAP scatterplot of scRNA-seq data (n = 23,519 cells) from^13^ re-annotated using markers from Fig. 1e. **e**: (Left and center) Integrated manifold (*in vivo* and *in vitro*) using scVI and coloured by cell state and specific conditions. Integration is performed with scVI. (Top right) Table displaying organoid-independent annotation for each scVI-integrated cluster. Organoid annotation matching *in vivo* labels displayed in green, discordant annotation in red. **f**: Violin plot showing normalised and log-transformed expression of differentially expressed genes (DEGs, limma, FDR < 0.05, with Bonferroni correction for multiple hypotheses testing) when comparing early EVT-2 in PTO vs *in vivo* EVT-2. (Right) Violin plot showing normalised and log-transformed expression of DEG when comparing iEVT in TSC vs *in vivo* iEVT. Primary trophoblast organoids (PTO), trophoblast stem cells (TSC), trophoblast organoid media (TOM), syncytiotrophoblast (SCT), villous cytotrophoblast (VCT), cytotrophoblast cell column (CCC), proliferative (p), extravillous trophoblast (EVT), interstitial EVT (iEVT), giant cells (GC), endovascular EVT (eEVT).

We next explored whether the cell-intrinsic regulatory programmes that are triggered upon VCT-to-EVT differentiation are also present in EVTs derived from both self-renewing PTOs^3^ and TSCs^5^. To do so, we performed scRNA-seq on: (1) PTOs differentiated in the presence of EVT medium (EVTM) (PTO-EVTM). PTOs grown in trophoblast organoid medium (TOM) (PTO-TOM) are used as controls; (2) TSCs differentiated in the presence of EVTM (TSC-EVTM). Here TSCs in trophoblast stem cell medium (TSCM) (TSC-TSCM) are used as controls (Fig. 3a and Extended Data Fig. 9a). In addition, to capture multinucleated SCT, we performed snRNA-seq on organoids grown in TOM and derived from both (3) PTOs and (4) TSCs (Extended Data Fig. 9a). We annotated the in vitro data using canonical trophoblast markers, transferring labels from the in vivo dataset into the in vitro dataset and integrating both in vivo and in vitro datasets on the same manifold (Fig. 3b and Extended Data Figs. 9b–l and 10a–e).

Projection of in vivo data onto in vitro trophoblasts using a logistic regression classifier that we trained on the in vivo dataset showed that VCT heterogeneity is better recapitulated in PTOs than in TSCs (Fig. 3c and Extended Data Fig. 10b). The four VCT subsets defined in vivo are present in PTOs and they express the same canonical markers (Fig. 3d). In PTOs, VCT-CCCs are enriched in the presence of EVTM, which triggers upregulation of the *FOXM1–NOTCH1* axis, NF-κB (*NFKB2* and *BACH2*) and AP-1 modulators ( *JDP2* and *ATF3*) (Fig. 3e and Extended Data Fig. 10d). By contrast, bona fide VCTs are not found among TSCs (Fig. 3c). Instead, when grown in TSCM, cells that we call ‘trophoblast stem cells’ (VCT-TSC) are primed to become VCT-CCCs as they upregulate VCT-CCC markers (*NOTCH1* and *ITGA2*) and downregulate some of the canonical VCT markers (*TP63*, *WLS*, *TNIK*, *SEMA3F*, *NRP2* and *BCAM*) (Fig. 3d). In both TSCs and PTOs, VCT-CCCs (*CDH5*, *ITGB6* and *LCAT1*) are enriched in the presence of EVTM media, which triggers the *NOTCH–FOXM1* axis, leading to a further downregulation of *IRF6* and *TP63*^23,31^ in EVT-1 (Fig. 3e,f). VCT-fusing is present in both PTOs and TSCs and accurately recapitulates its in vivo counterparts (Fig. 3c,d). snRNA-seq allowed us to capture mature SCT in PTOs (Extended Data Fig. 9d), and SCT in TSCs do not express *MFSD2A* (Extended Data Fig. 9i). Thus, our results highlight that the VCT subsets are accurately recapitulated in PTO, whereas bona fide VCTs are not found in TSCs.

VCT-CCCs in both PTOs and TSCs give rise to invasive EVT (EVT-1, EVT-2 early, EVT-2 and iEVT), whereas markers characteristic of GCs (high expression of *PRG2* and *AOC1*) and eEVT (*FLT4*, *NCAM1*, *GGT1*, *PPFIA4*, *MMP12* and *EIF4E1B*) are absent in our cultures (Extended Data Fig. 10a). Despite a good representation of almost all trophoblast subsets in both in vitro models, the relative proportion and efficiency of EVT differentiation was variable (Extended Data Fig. 9g,l). Similar to in vivo EVTs, EVTs derived from PTOs or TSCs downregulate the Wnt signalling pathway (*AXIN2*), upregulate members of the TGFβ signalling pathway (*TGB1*, *TGBR1* and *TGFBR2*) and express EVT markers (*ITGA1*, *PLAC8* and *HLA-G*) (Extended Data Fig. 10a,b). Markers of deep EVT invasion (*ERBB2*, *SERPINE1*, *SERPINE2* and *PAPPA*) are upregulated in iEVTs generated in PTOs or in TSCs. However, some differences in EVT states are found between in vivo and the two in vitro trophoblast models. For PTO there is an expansion of VCT-CCCs and an early EVT-2 that upregulates markers of both VCT-CCCs (*CDH5* and *LPCAT1*) and EVTs (*CSH1*, *FBLN1*, *TIMP3*, *CD81* and *EBI3)* when compared to in vivo EVT-2 (Extended Data Fig. 10d–f). By contrast, TSC captures an early iEVT state that is assigned as EVT-2 by our logistic regression model despite upregulating iEVT markers (Fig. 3c and Extended Data Fig. 10a,c). In line with this, TSC-iEVT-early clusters together with in vivo iEVTs but expresses lower levels of invasive markers (*SERPINE2*, *PLAC8*, *HLA-G* and *RAC1*) than its in vivo counterparts (Extended Data Fig. 10d–f). Altogether, major EVT invasion programmes are conserved in both PTOs and TSCs, yet there is an expansion of an early EVT population (EVT-2 early) in PTO and a less mature iEVT-like cluster is found in TSCs. The absence of deep invasive GCs and eEVTs in these cultures suggests that maternal cues present in vivo, specifically factors from the decidual stroma or maternal arteries and blood, respectively, are essential for generating these EVT end points.

## Maternal cells and EVT differentiation

**Fig. 4:**
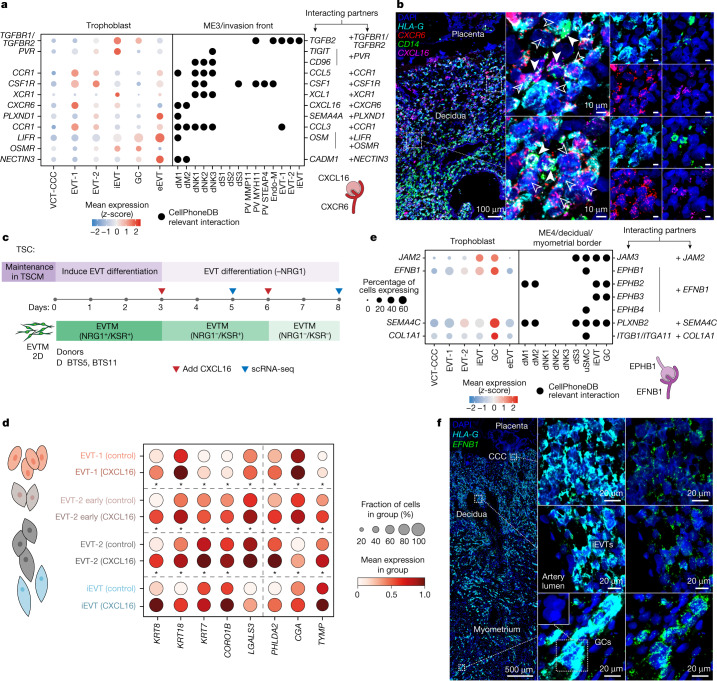
Predicted ligand–receptor interactions during EVT invasion. **a**, Left, dot plot showing *z*-score of normalized, log-transformed and variance-scaled gene expression of selected receptors (*y*-axis) that are upregulated in EVT-1, EVT-2 and/or iEVT (ME3) (*x*-axis). Right, dot plot showing the presence of selected ligands (*y*-axis) in cells present in ME3 (invasion front; *x*-axis). Differential expression as in Extended Data Fig. 8a. Schematic in bottom right represents select ligand–receptor interactions. **b**, Left, high-resolution multiplexed smFISH of placenta–decidua interface showing *HLA-G* (EVT) and *CD14* (decidual macrophage), and *CXCL16* and its cognate receptor *CXCR6*. Dashed outlines indicate areas shown magnified on the right. Centre, filled and unfilled arrows indicate neighbouring *CXCL16*-expressing decidual macrophages and *CXCR6*-expressing EVTs, respectively. Images are representative of two donors. **c**, Schematic representation of the EVT differentiation experimental design, indicating time points and biological replicates in TSC models (*n* = 2 donors). **d**, Dot plot showing normalized, log-transformed and variance-scaled expression of genes (*x*-axis) that are significantly upregulated (limma, FDR < 0.05, with Bonferroni correction for multiple hypotheses testing) in the EVT subsets upon exposure to CXCL16 compared with control. **e**, Left, dot plot showing *z*-score of normalized, log-transformed and variance-scaled gene expression of selected receptors (*y*-axis) that are upregulated in GC (ME4) (*x*-axis). Right, dot plot showing the presence (*y*-axis) of selected ligands in cells present in ME4 (decidual–myometrial border; *x*-axis). Differential expression as in Extended Data Fig. 8a. Schematic in bottom right represents select ligand–receptor interactions. **f**, High-resolution multiplexed smFISH of the placenta–decidua interface showing *HLA-G* and *EFNB1*, demonstrating that expression of *EFNB1* is present throughout EVTs, including iEVTs, and higher in GCs. The inset (bottom centre) illustrates the multinucleated nature of GCs. Images representative of two donors. dM, decidual macrophages; dS, decidual stromal cells; endo-M, maternal endotheial cells; PV, perivascular.

**Extended Data Fig. 11:**
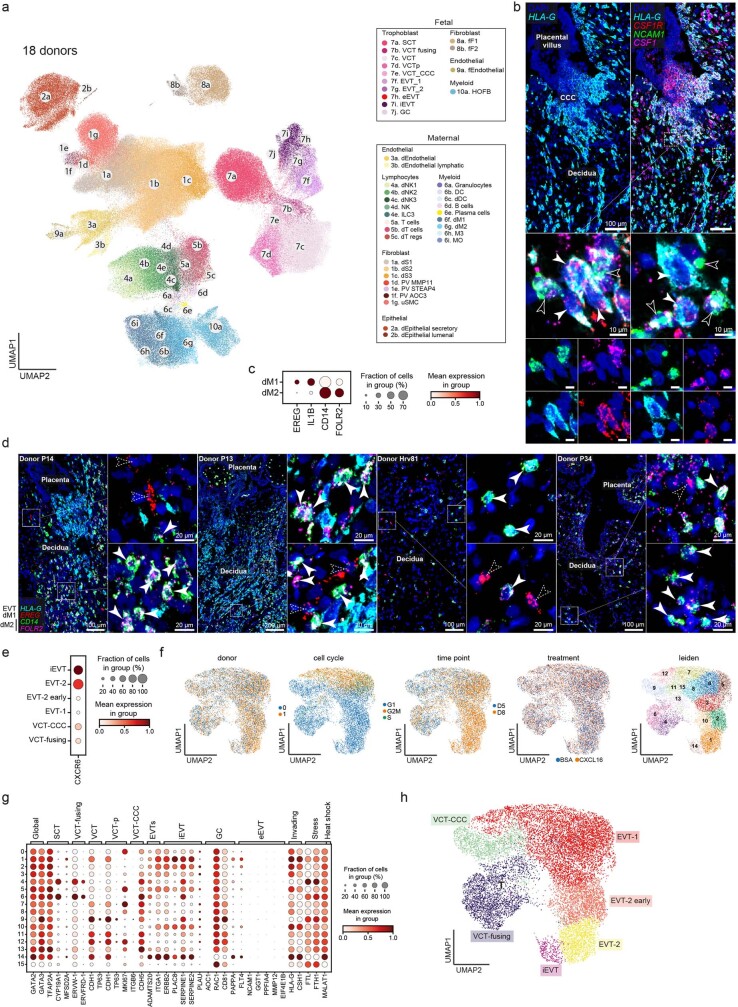
Predicted interactions between trophoblast and maternal immune cells. **a**: UMAP (uniform manifold approximation and projection) scatterplot of single-cell RNA sequencing (scRNA-seq) and single-nuclei RNA sequencing (snRNA-seq) data of the 18 donors described in Extended Data Fig. 1c of the maternal-fetal interface (n = 325,665 cells and nuclei) coloured by cell state. Integration was performed with scVI. **b**: (Left) High-resolution imaging of a section of the placenta-decidua interface stained by smFISH for *HLA-G*, highlighting EVTs invading the decidua from the CCC. (Right) multiplexed co-staining with *NCAM1* (dNK marker), *CSF1* and cognate receptor *CSF1R*; dashed squares indicate areas shown magnified to right. (Bottom) solid and outlined arrows indicate neighbouring *CSF1R*-expressing EVTs and *CSF1*-expressing dNK cells, respectively. Representative image of samples from three donors. **c**: Dot plot showing normalised, log-transformed and variance-scaled gene expression of macrophage markers (X-axis) in data from (a) (Y-axis). **d**: High-resolution imaging of the placenta-decidua interface stained by multiplexed smFISH for *HLA-G* (EVTs), *EREG* (dM1), and *CD14* and *FOLR2* (dM2) for n = 4 donors (donor ID is specified in each panel). **e**: Dot plot showing normalised, log-transformed and variance-scaled expression of *CXCR6* (X-axis) on the EVT subsets present in TSC (n = 2). **f**: UMAP scatterplots of scRNA-seq of TSC (CXCL16 and BSA conditions) coloured by donor, cell cycle phase, time point, treatment and unbiased clustering using leiden (n = 2). **g**: Dot plot showing normalised, log-transformed and variance-scaled expression of marker genes of the main trophoblast subsets (X-axis) in cell clusters defined in (f) (Y-axis) from the integrated manifold of CXCL16 and BSA conditions in trophoblast stem cell (TSC) scRNA-seq (n = 2). **h**: UMAP scatterplot of scRNA-seq of TSC coloured by cell state (n = 2). Villous cytotrophoblast (VCT), cytotrophoblast cell column (CCC), proliferative (p), extravillous trophoblast (EVT), interstitial EVTs (iEVTs), giant cells (GC), endovascular EVT (eEVT), dendritic cells (DC), lymphatic (l), maternal (m), fetal (f), Hofbauer cells (HOFB), innate lymphocytes (ILC), macrophages (M), monocytes (MO), natural killer (NK), perivascular (PV), decidual (d), epithelial (epi), stromal (S), fibroblasts (F), uterine smooth muscle cells (uSMC), bovine serum albumin (BSA).

We integrated single-cell and single-nuclei transcriptomics data from 18 donors to study how decidual maternal cells affect trophoblast invasion (Extended Data Figs. 1c,  2e and  11a). We used CellPhoneDB v4^37^ to determine the ligand–receptor interactions that are enriched in the four decidual microenvironments (Fig. 1a and Methods). We first focused on interactions mediating trophoblast invasion (Fig. 4a). As previously described^13^, decidual natural killer (dNK) cells interact with EVTs through multiple ligand–receptor pairs (PVR–TIGIT, PVR–CD96, CCR1–CCL5 and CSF1R–CSF1). We find that the majority of these receptors are upregulated in EVT-2, near the CCCs (Fig. 4a). In this location, the CSF1–CSF1R interaction is enriched, confirming previous findings^13,38^, and we reinforce this result using high-resolution multiplexed smFISH, which shows the close proximity of *CSF1*^+^ dNK cells and *CSF1R*^+^ EVT cells (Extended Data Fig. 11b).

We predicted multiple interactions between invading trophoblast cells and dM1 (*EREG*^*+*^ and *IL1B*^*+*^) and dM2 (*FOLR2*^*+*^ and *CD14*^high^) (Fig. 4a and Extended Data Fig. 11c,d). Maternal macrophages upregulate adhesion receptor genes, including *CADM1* (expressed in dM1 and dM2) and *SEMA4A* (expressed in dM1), whose cognate receptors *NECTIN3* and *PLXND1* are expressed in EVTs (Fig. 4a). In addition, both dM1 and dM2 express the chemokine genes *CXCL16* and *CCL3*, and their receptor genes *CXCR6*^39^ and *CCR1* are upregulated in invading EVTs (Fig. 4a). *CXCR6*^+^*HLA-G*^+^ EVTs and *CXCL16*^+^*CD14*^*+*^ decidual macrophages are in close proximity in the implantation site (Fig. 4b). Similar to their in vivo counterparts, scRNA-seq confirms that TSC-EVTs express *CXCR6*, and we used this model to functionally validate the effect of CXCL16 on EVTs (Fig. 4c and Extended Data Fig. 11e–h). CXCL16 upregulates the expression of characteristic placental genes (*PHLDA2* and *CGA*), those involved in endothelial integrity (*TYMP*) as well as cytokeratins (*KRT7*, *KRT8* and *KRT18*), actin-binding molecules (*CORO1B*) and the galectin member *LGALS3*, previously assigned to have a role in EVT invasion^40^ (Fig. 4d). This is in keeping with a role for CXCL16 in promoting trophoblast motility and function.

The receptors that are potentially involved in EVT invasion, including *CXCR6*, *CSF1R*^38^ and *PLXND1*, are downregulated in GCs (Fig. 4a), in keeping with their presence at the limit of EVT invasion^41^. GCs form by the fusion of iEVTs and upregulate adhesion genes (*JAM2*, *EFNB1* and *SEMA4C*) whose cognate receptor genes are expressed by other iEVTs (*JAM3*, *EPHB2*, *EPHB3* and *PLXNB2*), providing potential mechanisms for fusion (Fig. 4e). A possible explanation for iEVT migration from decidua into myometrium is the specific expression of EPHB1 and EPHB4^10^ by myometrial smooth muscle cells (uSMCs) which bind to EFNB1, which is upregulated in the iEVTs and GCs (Fig. 4e). We validated expression of *EFNB1* in GCs using multiplexed smFISH (Fig. 4f).

## eEVT interactions with spiral arteries

**Fig. 5:**
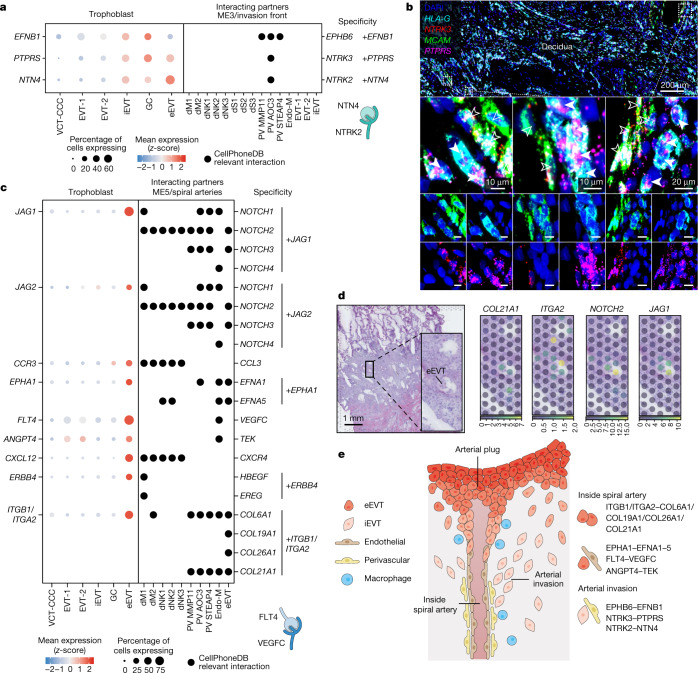
Predicted ligand–receptor interactions modulating uterine arterial transformation. **a**, Left, dot plot showing *z*-score of normalized, log-transformed and variance-scaled gene expression of selected receptors (*y*-axis) that are upregulated in iEVT (*x*-axis). Right, dot plot showing the presence of selected ligands (*y*-axis) in cells present in ME3 (invasion front; *x*-axis). Differential expression as in Extended Data Fig. 8a. **b**, Top, high-resolution smFISH of decidua stained for *HLA-G* and *MCAM* (PV marker), and *NTRK3* and its receptor *PTPRS*. Dashed outlines indicate areas that are shown magnified below. Middle and bottom, filled and unfilled arrows indicate neighbouring *PTPRS*-expressing EVTs and *NTRK3*-expressing dNK cells, respectively. Images are representative of three donors. **c**, Left, dot plot showing *z*-score normalized, log-transformed and variance-scaled gene expression of selected receptors (*y*-axis) that are upregulated in eEVT (*y*-axis). In the case of a complex, the expression corresponds to the least expressed subunit of the complex (*ITGB1*). Right, dot plot showing the presence of selected ligands (*y*-axis) in cells present in ME5 (spiral arteries; *x*-axis). Differential expression as in Extended Data Fig. 8a. **d**, Overview of spatial locations of invading trophoblast cell states in Visium spatial transcriptomics data of a representative section of donor P13 tissue. The position of the capture area is indicated with an arrow in Extended Data Fig. 1d. Spot colour indicates cell state densities computed by cell2location as the number of cells of a given cell state in a Visium spot. **e**, Schematic representation of the spiral arteries in the first trimester of human pregnancy, highlighting the novel interactions between PV–iEVT, endothelial–eEVT, and eEVT–eEVT.

**Extended Data Fig. 12:**
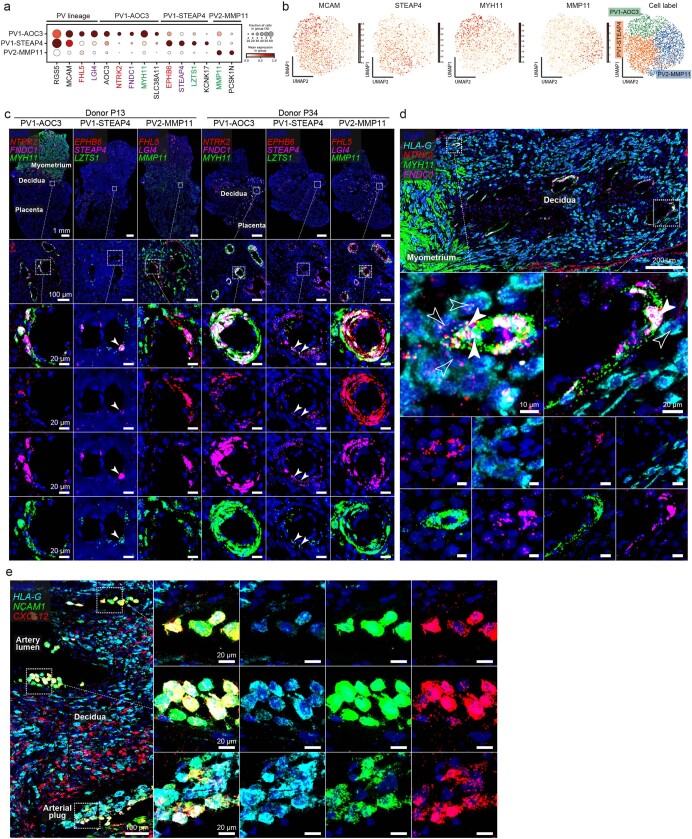
Interactions between trophoblast and perivascular (PV) cells. **a**: Dot plot showing normalised, log-transformed and variance-scaled expression of perivascular (PV) cell state markers. **b**: UMAP (uniform manifold approximation and projection) scatterplot of scRNA-seq of PV cells (n = 2768 cells) coloured by the scaled gene expression of PV cell state markers. **c**: (Top) High-resolution imaging of adjacent sections of maternal-fetal interface stained by multiplexed smFISH for three gene panels, from two donors. Dashed squares indicate areas shown magnified underneath (middle and below), highlighting PV1-*AOC3*, PV1-*STEAP4*, and PV2-*MMP11* cells expressing each of their three respective marker genes. Solid arrows indicate relatively sparse PV1-*STEAP4* cells in second and fifth columns. **d**: (Top) High-resolution imaging of a section of decidua stained by smFISH for *HLA-G* (EVTs) multiplexed with *MYH11*, *FNDC1*, and *NTRK2* (PV1-AOC3); dashed squares indicate areas shown magnified below. (Middle) solid and outlined arrows indicate neighbouring PV1-AOC3 cells expressing *NTRK2* and EVTs, respectively. Representative image of samples from two donors. **e**: (Left) High-resolution imaging of a section of decidua stained by multiplexed smFISH for *HLA-G*, *NCAM1*, and *CXCL12*. Dashed squares highlight arteries containing *HLA-G*+ *NCAM1*+ eEVTs expressing *CXCL12*, shown magnified to right. Representative image of samples from two donors.

Trophoblast arterial transformation during early pregnancy is crucial for pregnancy success. Initially, there is destruction of the media by iEVTs which is replaced with acellular fibrinoid material^1,27,41^. We previously defined two perivascular cell states^13^, PV1 (*MCAM*-high) and PV2 (*MMP11*-high) in the arterial media. Here we combine scRNA-seq and smFISH to identify two cell states within PV1: PV1-*AOC3* (*AOC3*-high, *MYH11*-high, *FNDC1*-high and *NTRK2*-high*)* and PV1-*STEAP4 (STEAP4*-high, *EPHB6*-high and *LZTS1*-high*)* (Extended Data Fig. 12a–c). We mapped the interactions between perivascular cell subsets and iEVT that might lead to medial destruction. Expression of *EFNB1* by iEVTs could induce their tropism towards the arteries as perivascular cells express the cognate receptor gene, *EPHB6* (Figs. 4f and  5a). We also find that iEVTs upregulate specific cell signalling molecules (*PTPRS* and *NTN4*) whose cognate receptor genes (*NTRK2* and *NTRK3*) are upregulated in PV1-*AOC3* (Fig. 5a). Neurotrophic tyrosine receptor kinases (NTRKs) can be associated with cellular survival. Whether they are involved in the ‘fibrinoid change’ in the arterial media^1,27,41,42^ would require further exploration. Using multiplexed smFISH, we validated the close proximity between iEVTs (*HLA-G*^+^*)* expressing *PTPRS* and perivascular cells (*MCAM*^+^) expressing *NTRK2* and *NTRK3* (Fig. 5b and Extended Data Fig. 12d).

eEVTs initially form plugs in the spiral arteries close to the cytotrophoblast shell that limit high-pressure maternal blood flow into the intervillous space before 8–10 PCW, prior to the establishment of the haemochorial circulation^43^. eEVTs eventually replace the maternal endothelium^41,42^. Our unbiased analyses enable us to speculate how the plugs are formed. In addition to the homotypic interactions by NCAM1, eEVTs express both *ITGB1* and *ITGA2* (forming the integrin α2β1) and its cognate collagen ligands (*COL6A1*, *COL19A1*, *COL26A1* and *COL21A1*) (Fig. 5c). Active Notch signalling is suggested by upregulation of ligand ( *JAG1* and *JAG2*) and receptor (*NOTCH2* and *NOTCH3*) genes (Fig. 5c). Interactions of eEVTs in the vasculature (ME5) could be mediated by *EPHA1*, *CXCL12*, *FLT4* and *ANGPT4*, with endothelial cells expressing their interacting partners *EFNA1*, *EFNA5*, *VEGFC* and *TEK* (Fig. 5c and Extended Data Fig. 12e). Using spatial transcriptomics, we visualized the expression of extracellular matrix (ECM) component (*COL21A1–ITGA2*) and Notch (*NOTCH2–JAG1*) interactions in the arterial plug (Fig. 5d).

Together, our high-resolution analyses of the spiral arteries in the decidua basalis enabled us to detect several ECM components and ligand–receptor pairs that are expressed in eEVT and maternal endothelial cells as well as in iEVT and PV subsets (Fig. 5e). These ligand–receptor interactions that occur between maternal and fetal cells are likely to be pivotal in mediating the maternal arterial transformation that is characteristic of the first trimester of pregnancy and is essential for its success.

## Discussion

In the postimplantation embryo, trophectoderm differentiates into trophoblast that invades the uterus to transform the maternal arteries. Defective trophoblast invasion is the primary underlying cause of the great obstetric syndromes that include pre-eclampsia, fetal growth restriction, unexplained stillbirth, placental abruption and preterm labour^2^. We made use of a historical collection of first-trimester pregnant hysterectomies to delineate the trophoblast landscape at the implantation site, where fetal and maternal cells intermingle. The human implantation sites profiled in our study were collected more than 30 years ago and have been stored in liquid nitrogen. We report new high-quality multiomics and spatial data, and developed a statistical framework (StOrder) that describes the complete trophoblast invasion trajectory during the first trimester of pregnancy. This includes the unbiased transcriptomics profile of eEVTs that replace the endothelium from the maternal arteries and placental bed GCs, present deeper in the decidua and the inner third of the myometrium. We use the complete in vivo trophoblast trajectory to benchmark current PTOs and TSCs in vitro trophoblast models and demonstrate that they faithfully recapitulate EVT differentiation. Terminal eEVTs and deep invasive GCs are absent in our in vitro cultures, and we reason that maternal signals from uterine cells and maternal serum are required to generate them.

Our systems biology approach has enabled us to explore potential interactions between EVTs and maternal decidual cells. First, we predict the ligand–receptor interactions between the maternal macrophages and EVT, in keeping with the importance of decidual innate immune cells for placentation^32^. We further explore the poorly described macrophage–EVT signalling axis in vitro and describe upregulation of motility genes in the EVT subsets. Second, we pinpoint the potential molecular and cellular mediators of arterial transformation during early pregnancy. Interactions between PV1-*AOC3* and iEVT could drive iEVT tropism towards the arterial wall and mediate the destruction of arterial smooth muscle media. eEVTs have a specific ECM that could allow them to form the plug. There are also specific interactions with endothelial cells that enable eEVTs to adhere to them. These novel interactions add to our understanding of the communication between endothelial and eEVT cells^44^. The effect of defective arterial transformation in the later stages of pregnancy is well-described and underpins the great obstetric syndromes^9^. Our study increases the understanding of these major pregnancy disorders, all of which have their origins in the first trimester^45^. In addition, our roadmap of trophoblast differentiation can be used as a blueprint to design improved in vitro models that fully recapitulate the early stages of implantation.

## Methods

### Human samples

Placental and decidual samples used for the in vivo and in vitro profiling were obtained from elective terminations from: The MRC and Wellcome-funded Human Developmental Biology Resource (HDBR, https://www.hdbr.org), with appropriate maternal written consent and approval from the Fulham Research Ethics Committee (REC reference 18/LO/0822) and Newcastle and North Tyneside 1 Research Ethics Committee (REC reference 18/NE/0290). The HDBR is regulated by the UK Human Tissue Authority (HTA; https://www.hta.gov.uk) and operates in accordance with the relevant HTA Codes of Practice.Addenbooke’s Hospital (Cambridge) under ethical approval from the Cambridge Local Research Ethics Committee (04/Q0108/23), which is incorporated into the overarching ethics permission given to the Centre for Trophoblast Research biobank for the Biology of the Human Uterus in Pregnancy and Disease Tissue Bank at the University of Cambridge under ethical approval from the East of England-Cambridge Central Research Ethics Committee (17/EE/0151) and from the London-Hampstead Research Ethics Committee (20/LO/0115).

Placental–decidual blocks (P13, P14 and P34) were collected prior to 1 September 2006 and consent for research use was not obtained. These samples are considered ‘Existing Holdings’ under the Human Tissue Act and as such were able to be used in this project. All the other tissue samples used for this study were obtained with written informed consent from all participants in accordance with the guidelines in The Declaration of Helsinki 2000.

All samples profiled were histologically normal.

TSC lines BTS5 and BTS11 derived from human blastocysts by H. Okae and colleagues^5^ were used in this study. Informed consent was obtained from all donors prior to the establishment of the cell line and the study was approved by the Ethics Committee of Tohoku University School of Medicine (Research license 2016-1-371), associated hospitals, the Japan Society of Obstetrics and Gynecology and the Ministry of Education, Culture, Sports, Science and Technology (Japan). This work was internally approved by HuMFre-20-0005 at the Wellcome Sanger Institute and the lines were covered by a Conditions of Use agreement with the Tohoku University School of Medicine (internal reference CG175).

### Tissue cryopreservation

Fresh tissue samples of human implantation sites were embedded in cold OCT medium and flash-frozen using a dry ice-isopentane slurry as described^46^.

Quality of archival frozen tissue samples was assessed by extraction of RNA from cryosections using the QIAGEN RNeasy Mini Kit, according to the manufacturer’s instructions including on-column DNase I digestion. RNA quality was assayed using the Agilent RNA 6000 Nano Kit. All samples processed for Visium and single-nuclei had RIN values greater than 8.7.

### Single-nuclei extraction

Single-nuclei suspensions were isolated from frozen tissue sections when performing multiomic snRNA-seq, scATAC-seq and snRNA-seq, following the manufacturer’s instructions. For each OCT-embedded sample, 400 μm of tissue was prepared as 50 μm cryosections, which were paused in a tube on dry ice until subsequent processing. Nuclei were released via Dounce homogenization as described^47^.

### Single-cell isolation from tissue

We used the previous protocol optimized for the decidual–placental interface^13^. In short, decidual tissues were enzymatically digested in 15 ml 0.4 mg ml^−1^ collagenase V (Sigma, C9263) solution in RPMI 1640 medium (Thermo Fisher Scientific, 21875-034)/10% FCS (Biosfera, FB-1001) at 37 °C for 45 min. The supernatant was diluted with medium and passed through a 100-μm cell sieve (Corning, 431752) and then a 40-μm cell sieve (Corning, 431750). The flow-through was centrifuged and resuspended in 5 ml of red blood cell lysis buffer (Invitrogen, 00-4300) for 10 min. Placental villi were scraped from the chorionic membrane using a scalpel and the stripped membrane was discarded. The resultant villous tissue was enzymatically digested in 70 ml 0.2% trypsin 250 (Pan Biotech P10-025100P)/0.02% EDTA (Sigma E9884) in PBS with stirring at 37 °C for 9 min. The disaggregated cell suspension was diluted with medium and passed through a 100-μm cell sieve (Corning, 431752). The undigested gelatinous tissue remnant was retrieved from the gauze and further digested with 10–15 ml collagenase V at 1.0 mg ml^−1^ (Sigma C9263) in Ham’s F12 medium/10% FBS with gentle shaking at 37 °C for 10 min. The disaggregated cell suspension was diluted with medium and passed through a 100 μm cell sieve (Corning, 431752). Cells obtained from both enzyme digests were pooled together and passed through a 100 μm cell sieve (Corning, 431752) and washed in Ham’s F12. The flow-through was centrifuged and resuspended in 5 ml of red blood cell lysis buffer (Invitrogen, 00-4300) for 10 min.

### Trophoblast in vitro cultures

Trophoblast stem cell (TSC) lines BTS5 and BTS11 derived by Okae and colleagues were grown as described previously^5^. In brief, TSC self-renewing medium (TSCM) components were substituted with local suppliers with the exception for 30% w/v BSA from WAKO Japan and CHIR99021 concentration was increased to 6 µM which maintained the undifferentiated morphology as well as preserving its EVT invasive morphology. TSCs were grown on 5 µg ml^−1^ Collagen IV (Corning) coated wells and early passaged cells between passages 24 and 26 were used for differentiation and analysis. For 2D differentiation into EVT identity, cells were seeded at a density of 1.3 × 10^5^ per cm^2^ (corresponding to 125,000 cells plated on a well of a 6-well plate) in EVTM1 detailed below supplemented with ice-cold 2% Matrigel GFR (Corning) before seeding on 1 µg ml^−1^ Collagen IV (Corning) coated wells (D0). Three days later (D3), medium was changed to EVTM2 supplemented with ice-cold 0.5% Matrigel GFR. Three days later (D6), the medium was changed to EVT medium 3 supplemented with ice-cold 0.5% Matrigel GFR. Cells were treated with TrypLE for downstream analysis 48 h later (D8). For CXCL16 induction experiments, a final concentration of 100 ng ml^−1^ CXCL16 (RnD 976-CX-025 with carrier, dissolved in 0.1%BSA(WAKO)/PBS) were supplemented to EVTM2 or EVTM3 and analysed 48 h later. The induction was controlled by supplementing an equal volume of 0.1% BSA/PBS.

In total, six trophoblast organoids were grown and differentiated into EVT as previously described^3,48^. To differentiate trophoblast organoids into EVT, organoids were cultured with TOM for ~3–4 days and transferred into EVTM1 (+NRG1) for ~4–7 days. Once trophoblasts initiate their commitment into EVT (spike emergence), EVTM2 (−NRG1) is added for 4 days. Donors were differentiated and collected in batches of three that were multiplexed on the same 10x Genomics reaction. Samples for donors 1, 2 and 3 were collected at 3 h, 24 h and 48 h after the addition of EVTM2, while samples for donors 4, 5 and 6 were collected at 48 h before, and then 0 h, 48 h and 96 h after, addition of EVTM2. Organoids grown in TOM were also collected as a control at 96h.

Media compositions have been described previously^3,5,48^ and are shown here. TSCM: DMEM/F12 with Glutamax (Gibco) supplemented with 0.2% v/v FBS (Gibco), 0.3% wt/vol BSA (WAKO), 1% ITS-X (Gibco), 2.5 µg ml^−1^ l-ascorbic acid-2-phosphate (Sigma), 50 ng ml^−1^ EGF (Peprotech AF-100-15), 6 µM CHIR99021 (Tocris 4423), 0.5 µM A83-01 (Tocris 2939), 1 µM SB43154 (Tocris 1614), 0.8 mM VPA (Sigma, dissolved in H2O) and 5 µM Y-27632 (Millipore 688000). TOM: Advanced DMEM/F12, N2 supplement (at manufacturer’s recommended concentration), B27 supplement minus vitamin A (at manufacturer’s recommended concentration), Primocin 100 μg ml^−1^, *N*-Acetyl-l-cysteine 1.25 mM, l-glutamine 2 mM, recombinant human EGF 50 ng ml^−1^, CHIR99021 1.5 µM, recombinant human R-spondin-1 80 ng ml^−1^, recombinant human FGF-2 100 ng ml^−1^, recombinant human HGF 50 ng ml^−1^, A83-01 500 nM, prostaglandin E2 2.5 µM, Y-27632 5 µM. EVTM1: Advanced DMEM/F12 (or DMEM/F12 for TSC-EVTM 2D), l-glutamine 2 mM, 2-mercaptoethanol 0.1 mM, penicillin/streptomycin solution 0.5% (vol/vol), BSA 0.3% (wt/vol, WAKO), ITS-X supplement 1% (vol/vol), NRG1 (Cell Signaling 5218SC) 100 ng ml^−1^, A83-01 7.5 µM, knockout serum replacement 4% (vol/vol). EVTM2, Advanced DMEM/F12 (or DMEM/F12 for TSC-EVTM 2D), l-glutamine 2 mM, 2-mercaptoethanol 0.1 mM, penicillin/streptomycin solution 0.5% (vol/vol), BSA 0.3% (wt/vol, WAKO), ITS-X supplement 1% (vol/vol), A83-01 7.5 µM, Knockout serum replacement 4% (vol/vol) (this is the same as EVTM1 without NRG1). This medium can be stored at 4 °C for up to 1 week. EVTM3, DMEM/F12 (for TSC-EVTM 2D), l-glutamine 2 mM, 2-mercaptoethanol 0.1 mM, penicillin/streptomycin solution 0.5% (vol/vol), BSA 0.3% (wt/vol, WAKO), ITS-X supplement 1% (vol/vol), A83-01 7.5 µM (this is the same as EVTM1 without NRG1 or knockout serum replacement). This can be stored at 4 °C for up to 1 week.

### H&E staining and imaging

Fresh frozen sections were removed from −80 °C storage and air dried before being fixed in 10% neutral buffered formalin for 5 min. After rinsing with deionised water, slides were stained in Mayer’s haematoxylin solution for 90 s. Slides were completely rinsed in 4–5 washes of deionised water, which also served to blue the haematoxylin. Aqueous eosin (1%) was manually applied onto sections with a pipette and rinsed with deionised water after 1–3 s. Slides were dehydrated through an ethanol series (70%, 70%, 100%, 100%) and cleared twice in 100% xylene. Slides were coverslipped and allowed to air dry before being imaged on a Hamamatsu Nanozoomer 2.0HT digital slide scanner.

### Multiplexed smFISH and high-resolution imaging

Large tissue section staining and fluorescent imaging were conducted largely as described previously^49^. Sections were cut from fresh frozen samples embedded in OCT at a thickness of 10–16 μm using a cryostat, placed onto SuperFrost Plus slides (VWR) and stored at −80 °C until stained. Tissue sections were processed using a Leica BOND RX to automate staining with the RNAscope Multiplex Fluorescent Reagent Kit v2 Assay (Advanced Cell Diagnostics, Bio-Techne), according to the manufacturers’ instructions. Probes are listed in Supplementary Table 8. Prior to staining, fresh frozen sections were post-fixed in 4% paraformaldehyde in PBS for 6–8 h, then dehydrated through a series of 50%, 70%, 100%, and 100% ethanol, for 5 min each. Following manual pre-treatment, automated processing included heat-induced epitope retrieval at 95 °C for 15 min in buffer ER2 and digestion with Protease III for 15 min prior to probe hybridisation. Tyramide signal amplification with Opal 520, Opal 570, and Opal 650 (Akoya Biosciences) and TSA-biotin (TSA Plus Biotin Kit, Perkin Elmer) and streptavidin-conjugated Atto 425 (Sigma Aldrich) was used to develop RNAscope probe channels.

Stained sections were imaged with a Perkin Elmer Opera Phenix Plus High-Content Screening System, in confocal mode with 2 μm *z*-step size, using a 40× (NA 1.1, 0.149 μm/pixel) water-immersion objective. Channels: DAPI (excitation 375 nm, emission 435–480 nm), Atto 425 (excitation 425 nm, emission 463–501 nm), Opal 520 (excitation 488 nm, emission 500–550 nm), Opal 570 (excitation 561 nm, emission 570–630 nm), Opal 650 (excitation 640 nm, emission 650–760 nm).

### Image stitching

Confocal image stacks were stitched as two-dimensional maximum intensity projections using proprietary Acapella scripts provided by Perkin Elmer.

### 10x Genomics Chromium GEX library preparation and sequencing

For the scRNA-seq experiments, cells were loaded according to the manufacturer’s protocol for the Chromium Single Cell 3′ Kit v3.0, v3.1 and 5’ v1.0 (10X Genomics). Library preparation was carried out according to the manufacturer’s protocol to attain between 2,000 and 10,000 cells per reaction. Libraries were sequenced, aiming at a minimum coverage of 20,000 raw reads per cell, on the Illumina HiSeq 4000 or Novaseq 6000 systems using the following sequencing format: (A) read 1: 26 cycles; i7 index: 8 cycles, i5 index: 0 cycles; read 2: 98 cycles; (B) read 1: 28 cycles; i7 index: 8 cycles, i5 index: 0 cycles; read 2: 91 cycles; (C) read 1: 28 cycles; i7 index: 10 cycles; i5 index: 10 cycles; read 2: 90 cycles (v3.1 dual).

For the multimodal snRNA-seq and scATAC-seq experiments, cells were loaded according to the manufacturer’s protocol for the Chromium Single Cell Multiome ATAC + Gene Expression v1.0 to attain between 2,000 and 10,000 cells per well. Library preparation was carried out according to the manufacturer’s protocol. Libraries for scATAC-seq were sequenced on Illumina NovaSeq 6000, aiming at a minimum coverage of 10,000 fragments per cell, with the following sequencing format; read 1: 50 cycles; i7 index: 8 cycles, i5 index: 16 cycles; read 2: 50 cycles.

### 10x Genomics Visium library preparation and sequencing

Ten-micrometre cryosections were cut and placed on Visium slides, then processed according to the manufacturer’s instructions. In brief, sections were fixed with cold methanol, H&E stained and imaged on a Hamamatsu NanoZoomer S60 before permeabilization, reverse transcription and cDNA synthesis using a template-switching protocol. Second-strand cDNA was liberated from the slide and single-indexed libraries were prepared using a 10x Genomics PCR-based protocol. Libraries were sequenced (1 per lane on a HiSeq 4000), aiming for 300M raw reads per sample, with the following sequencing format; read 1: 28 cycles, i7 index: 8 cycles, i5 index: 0 cycles and read 2: 91 cycles.

### Alignment and quantification of scRNA-seq and snRNA-seq data

For each sequenced single-cell and single-nucleus RNA-seq library, we performed read alignment to the 10X Genomics’ GRCh38 3.0.0 human reference genome, mRNA version for scRNA-seq samples and pre-mRNA version for snRNA-seq samples, latter created following instructions from 10X Genomics: https://support.10xgenomics.com/single-cell-gene-expression/software/pipelines/latest/advanced/references#premrna. Quantification and initial quality control were performed using the Cell Ranger Software (version 3.0.2; 10X Genomics) using default parameters. Cell Ranger filtered count matrices were used for downstream analysis.

### Alignment and quantification of multiome data

For each sequenced snRNA-seq and ATAC–seq (multiome) library, we performed read alignment to custom made genome consisting of 10X Genomics’ GRCh38 3.0.0 pre-mRNA human reference genome and 10X Genomics Cell Ranger-Arc 1.0.1 ATAC genome, created following instructions from 10X Genomics: https://support.10xgenomics.com/single-cell-multiome-atac-gex/software/pipelines/latest/advanced/references. Quantification and initial quality control were performed using the Cell Ranger-Arc Software (version 1.0.1; 10X Genomics) using default parameters. Cell Ranger-Arc filtered count matrices were used for downstream analysis.

### Downstream scRNA-seq and snRNA-seq analysis

#### Detection of doublets by gene expression

We used Scrublet for cell doublet calling on a per-library basis. We used a two-step diffusion doublet identification followed by Bonferroni FDR correction and a significance threshold of 0.01, as described in^50^. Predicted doublets were not excluded from the initial analysis, but used afterwards to flag clusters with high doublet scores.

#### Detection of doublets by genotype

Souporcell^51^ was used to deconvolute (1) maternal and fetal origin of cells and nuclei in our scRNA-seq and snRNA-seq samples (including multiome snRNA-seq); (2) assignment of cells to individuals in pooled samples (namely, samples Pla_HDBR8768477, Pla_HDBR8715512 and Pla_HDBR8715514); and (3) organoids from multiple individuals. In some samples deconvolution into maternal or fetal origin by genotype was not possible which is probably owing to the highly skewed ratio of genotypes (either extremely high (>0.95) or extremely low (<0.05) ratio of maternal to fetal droplets). In those cases, maternal–fetal origin of the cells was identified using known markers from ref. ^13^.

Souporcell (version 2.4.0) was installed as per instructions in https://github.com/wheaton5/souporcell and used in the following way:

path_to/singularity exec ./souporcell.sif souporcell_pipeline.py -i ./cellranger_path/possorted_genome_bam.bam -b ./cellranger_path/filtered_feature_bc_matrix/barcodes.tsv -f ./genome_path/genome.fa -t 8 -o souporcell_result -k 2 --skip_remap True --common_variants ./filtered_2p_1kgenomes_GRCh38.vcf

Where *k* = 2 corresponds to the number of individuals to be deconvoluted (in our case either mother and fetus or pooled individuals H7 and H9 in samples Pla_HDBR8768477, Pla_HDBR8715512 and Pla_HDBR8715514. The accuracy of deconvolution was evaluated in downstream analysis once cluster identity was clear from either gene expression or predictions of logistic regression. In samples where deconvolution worked successfully, inter-individual doublets were further excluded from downstream analysis.

#### Filtering genes high in ambient RNA signal

To assess which genes in the scRNA-seq and snRNA-seq data were high in ambient RNA (soup) signal (further referred to as noisy genes), the following approach was undertaken separately for all the scRNA-seq and snRNA-seq samples: (1) Read in all the raw and filtered count matrices for each sample produced by Cell Ranger Software. (2) Discard droplets with < 5 unique moleular identifiers (UMIs) (likely to be fake droplets from sequencing errors). (3) Only keep data from samples which we further consider as noisy (where ‘Fraction reads in cells’ reported by Cell Ranger is less than 70% (guided by 10X Genomics’ recommendations: https://assets.ctfassets.net/an68im79xiti/163qWiQBTVi2YLbskJphQX/e90bb82151b1cdab6d7e9b6c845e6130/CG000329_TechnicalNote_InterpretingCellRangerWebSummaryFiles_RevA.pdf). (4) Take the droplets that are in raw but are not in filtered matrices considering them as empty droplets. (5) Concatenate all raw objects with empty droplets into 1 joint raw object and do the same for filtered. (6) For all genes calculate soup probability as defined with the following equation: $P={E}_{g}^{{\rm{empty}}\,{\rm{droplets}}}/({E}_{g}^{{\rm{empty}}\,{\rm{droplets}}}+{E}_{g}^{{\rm{cells}}/{\rm{nuclei}}})$, where ${E}_{g}^{{\rm{empty}}\;{\rm{droplets}}}$ is the total sum of expression (number of UMI counts) of gene *g* in empty droplets, and ${E}_{g}^{{\rm{cells}}/{\rm{nuclei}}}$ is the total sum of expression counts of gene *g* in droplets that are considered as cells/nuclei by Cell Ranger. (7) For all genes calculate number of cells/nuclei where the gene is detected at >0 expression level (UMI counts). (8) Label genes as noisy if their soup probability exceeds 50% quantile of soup probability distribution - done separately for cells and for nuclei.

This approach was used to estimate noisy genes in (1) donor P13 samples and (2) all donors’ samples. Donor P13 noisy genes were excluded during mapping onto space (Visium, see ‘Location of cell types in Visium data’), whereas all donors’ noisy genes (labelled using nuclei-only derived threshold in step 8 to not over-filter genes based on the higher quality portion of the data which in our case in scRNA-seq) were excluded during all donors analysis of the whole atlas of all the cell states at the maternal–fetal interface.

#### Quality filters, alignment of data across different batches and clustering

We integrated the filtered count matrices from Cell Ranger and analysed them with scanpy (version 1.7.1), with the pipeline following their recommended standard practises. In brief, we excluded genes expressed by less than three cells, excluded cells expressing fewer than 200 genes, and cells with more than 20% mitochondrial content. After converting the expression space to log(CPM/100 + 1), the object was transposed to gene space to identify cell cycling genes in a data-driven manner, as described in^50,52^. After performing principal component analysis (PCA), neighbour identification and Louvain clustering, the members of the gene cluster including known cycling genes (*CDK1*, *MKI67*, *CCNB2* and *PCNA*) were flagged as the data-derived cell cycling genes, and discarded in each downstream analysis where applicable.

Next, to have an estimate of the optimal number of latent variables to be used later in the single-cell variational inference (scVI) workflow for dimensionality reduction and batch correction, we identified highly variable genes, scaled the data and calculated PCA to observe the variance ratio plot and decide on an elbow point which defined values of n_latent parameter which were then used to correct for batch effect by 10X library batch (‘sample’) with scVI. Number of layers in scVI models was tuned manually to allow for better integration. The resulting latent representation of the data was used for calculating neighbourhood graph, UMAP and further Louvain clustering. For trophoblast organoid scRNA-seq and snRNA-seq, data were integrated with Harmony by donor using theta = 0 parameter.

Analysis was done separately for (a) donor P13 trophoblast compartment and (b) all donors’ data (all cell states). In both analyses (a) and (b) trophoblast data was analysed separately with consecutive rounds of re-analysis upon exclusion of clusters of noisy nature (exhibiting gene expression characteristic of more than 1 distinct population). In addition, in all donors’ analysis fibroblast (maternal and fetal separately) and maternal NK, T, myeloid, epithelial, endothelial and perivascular compartments were reanalysed separately using the approach described in the previous paragraph to achieve fine grain annotation.

### Differential gene expression analysis

Differential gene expression analysis was performed with limma (limma version 3.46.0, edgeR version 3.32.1) with “cell_or_nucleus” covariate (scRNA-seq or snRNA-seq (including multiome snRNA-seq) origin of each droplet) backwards along the trajectory that was derived using stOrder approach, namely for the following 6 comparisons: VCT-CCC vs VCT (VCT and VCT-p cell states together); EVT-1 vs VCT-CCC; EVT-2 vs EVT-1; iEVT vs EVT-2; GC vs iEVT; eEVT vs EVT-2. Only significant DEGs were considered for downstream analysis, namely those with FDR (bonferroni) < 0.05).

### Alignment, quantification and quality control of multiome ATAC data

We processed scATAC-seq libraries coming from multiome samples (read filtering, alignment, barcode counting, and cell calling) with 10X Genomics Cell Ranger-Arc (version 1.0.1) using the pre-built 10X GRCh38 genome (version corresponding to Cellranger-arc 1.0.1) as reference. We called the peaks using an in-house implementation of the approach described in Cusanovich et al. ^53^ (available at https://github.com/cellgeni/cellatac, revision 21-099). In short, the genome was broken into 5-kb windows and then each cell barcode was scored for insertions in each window, generating a binary matrix of windows by cells. Matrices from all samples were concatenated into a unified matrix, which was filtered to retain only the top 200,000 most commonly used windows per sample. Using Signac (https://satijalab.org/signac/ version 0.2.5), the binary matrix was normalized with term frequency-inverse document frequency (TF-IDF) followed by a dimensionality reduction step using Singular Value Decomposition (SVD). The first latent semantic indexing (LSI) component was ignored as it usually correlates with sequencing depth (technical variation) rather than a biological variation^53^. The 2–30 top remaining components were used to perform graph-based Louvain clustering. Next, peaks were called separately on each cluster using macs2^54^. Finally, peaks from all clusters were merged into a master peak set (that is, peaks overlapping in at least one base pair were aggregated) and used to generate a binary peak-by-cell matrix, indicating any reads occurring in each peak for each cell.

This analysis was done separately for (1) all multiome data at first and (2) trophoblast-only subset of the multiome data. In the latter analysis we used annotation labels from the RNA counterpart of the multiome samples to perform peak calling.

### Alignment, quantification and quality control of Visium data

For each 10X Genomics Visium sample, we used Space Ranger Software Suite (version 1.1.0) to align to the GRCh38 human reference pre-mRNA genome (official Cell Ranger reference, version 3.0.0) and quantify gene counts. Spots were automatically aligned to the paired H&E images by Space Ranger software. All spots under tissue detected by Space Ranger were included in downstream analysis.

### Downstream analysis of 10X Genomics Visium data

#### Location of cell types in Visium data

To locate the cell states in the Visium transcriptomics slides, we used the cell2location tool v0.06-alpha^55^. As reference, we used snRNA-seq data of donor P13. We used general cell state annotations from the joint all donors’ analysis (corresponding to donor P13 data), with the exception of the trophoblast lineage. Trophoblast annotations were taken from donor P13-only analysis of the trophoblast compartment. Using information about which genes are noisy (high in ambient RNA signal) in donor P13 snRNA-seq data (details in ‘Filtering genes high in ambient RNA signal’), we excluded those from the reference and Visium objects prior to cell2location model training which significantly improved the results of mapping (namely, eliminated off-target mapping of cell states—that is, made results of mapping more specific to the correct anatomical regions). Following the tutorial at https://cell2location.readthedocs.io/en/latest/notebooks/cell2location_tutorial.html#Cell2location:-spatial-mapping, we trained cell2location model with default parameters using 10X library as a batch covariate in the step of estimation of reference cell-type signatures. Results were visualized with scanpy (version 1.7.1). Plots represent estimated abundance of cell types (cell densities) in Visium spots.

#### Subsetting Visium data into anatomical regions with SpatialDE2

We used SpatialDE2^56^ tissue segmentation algorithm to assign Visium spots to three anatomical regions: (1) placenta; (2) decidua and villi tips; and (3) myometrium. We used mRNA abundances from the deconvolution results obtained with cell2location^17^ in SpatialDE2 tissue segmentation. Assignment of obtained Visium spot clusters to regions was done upon visual inspection. Locations of certain fibroblast cell states indicative of the specific anatomical region (uterine smooth muscle cells, uSMC and dS cell states) were also used to guide this assignment. In addition, low-quality spots were discarded on the basis of not being under tissue and having low count and gene coverage (visual inspection).

For more details, please refer to the following notebook: https://github.com/ventolab/MFI/blob/main/2_inv_troph_trajectory_and_TFs/2-1_stOrder_inv_troph/S1_regions_analysis_for_SpCov_model_and_later_for_CellPhone.ipynb

### Downstream snATAC-seq analysis

#### Quality filters

To obtain a set of high-quality peaks for downstream analysis, we filtered out peaks that (1) were included in the ENCODE blacklist, (2) have a width outside the 210–1,500 bp range, and (3) were accessible in less than 5% of cells from a cellatac cluster. Low-quality cells were also removed by setting to 4 the minimum threshold for log1p-transformed total counts per cell.

#### Alignment of data across different batches and clustering

We adopted the cisTopic approach^57,58^ for the core of our downstream analysis. cisTopic employs latent Dirichlet allocation (LDA) to estimate the probability of a region belonging to a regulatory topic (region–topic distribution) and the contribution of a topic within each cell (topic–cell distribution). The topic–cell matrix was used for constructing the neighbourhood graph, computing UMAP projections and clustering with the Louvain algorithm. After this was done for all cell states, clusters corresponding to trophoblast cell states (based on the unbiased clustering done here and annotation labels coming from the RNA counterpart of this multiome data) were further subsetted and reanalysed following the same pipeline.

#### Gene activity scores

Next, we generated a denoised accessibility matrix (predictive distribution) by multiplying the topic–cell and region–topic distribution and used it to calculate gene activity scores. To be able to integrate them with scRNA-seq and snRNA-seq data, gene activity scores were rounded and multiplied by a factor of 10^7^, as described^58^.

#### Cell-type annotation of invading trophoblast

Final labels of invading trophoblast in snATAC-seq data were directly transferred from RNA counterpart of the multiome data.

### Join inference of trophoblast invasion from gene expression and spatial data

StOrder is a computational framework for joint inference of cellular differentiation trajectories from gene expression data and information about location of cell states in physical space (further referred to as spatial data).

It consists of three principal steps:

1. Calculate pairwise cell state connectivity from gene expression data (here we use snRNA-seq data).
2. Calculate pairwise cell state proximity in physical space from spatial data (here we use Visium spatial transcriptomics data) using a new spatial covariance model.
3. Combine connectivity matrices from steps 1 and 2 in a weighted expression to reconstruct the putative tree structure of the differentiation trajectory.

First, StOrder relies on a gene expression-based connectivity matrix (generated in our case by PAGA^59^) that establishes potential connections between cell state clusters defined by single-cell or single-nucleus transcriptomics datasets. The values in this matrix can be interpreted as pairwise similarity scores for cell states in gene expression space. In our case we used snRNA-seq data from P13 as it contains all trophoblast subsets.

Second, StOrder generates a spatial covariance matrix that reflects pairwise proximity of cell states that co-exist in space and smoothly transition from one state to another while physically migrating in space. To do so, StOrder takes as an input the deconvolution results (derived in our case with cell2location^17^) of Visium spatial transcriptomics data. Here, we used all spatial transcriptomics data profiled (donors P13, P14 and Hrv43). Then, it fits a Gaussian process model that derives pairwise spatial covariance scores for all the cell state pairs with the following model:

$${\rm{vec}}({{\bf{Y}}}_{i},{{\bf{Y}}}_{j}) \sim {\mathscr{N}}\,\left(0,\,\left(\begin{array}{cc}{\sigma }_{1}^{(1)} & {\sigma }_{2}^{(1)}\\ {\sigma }_{2}^{(1)} & {\sigma }_{3}^{(1)}\end{array}\right)\otimes K({\bf{X}},l)+\left(\begin{array}{cc}{\sigma }_{1}^{(2)} & 0\\ 0 & {\sigma }_{2}^{(2)}\end{array}\right)\otimes {\bf{I}}\right)$$

where ⊗ is the Kronecker product and the combined vector of cell densities (**Y**_*i*,*k*_, **Y**_*j*,*k*_**)** of cell states *i* and *j* is modelled by a multivariate Gaussian distribution whose covariance decomposes into a spatial and a noise term. The spatial term

$$\left(\begin{array}{cc}{\sigma }_{1}^{(1)} & {\sigma }_{2}^{(1)}\\ {\sigma }_{2}^{(1)} & {\sigma }_{3}^{(1)}\end{array}\right)\otimes K\left({\bf{X}},l\right)$$

is defined by a between-cell-state covariance matrix

$$\left(\begin{array}{cc}{\sigma }_{1}^{(1)} & {\sigma }_{2}^{(1)}\\ {\sigma }_{2}^{(1)} & {\sigma }_{3}^{(1)}\end{array}\right)$$

and a spatial covariance matrix defined using the squared exponential kernel:

$$K{({\bf{X}},l)}_{mn}=\exp \left(-\frac{{\parallel {x}_{m}-{x}_{n}\parallel }^{2}}{2{l}^{2}}\right)$$

*x*_*m*_ and *x*_*n*_ are spatial coordinates of spots *m* and *n* and *l* is the length scale of the smooth Gaussian process function in space that is being fit to cell densities.

The noise term

$$\left(\begin{array}{cc}{\sigma }_{1}^{(2)} & 0\\ 0 & {\sigma }_{2}^{(2)}\end{array}\right)\otimes {\bf{I}}$$

represents sources of variation other than spatial covariance of cell state densities.

The between-cell-state covariance matrix is constrained to be symmetric positive definite by defining

$$\left(\begin{array}{cc}{\sigma }_{1}^{(1)} & {\sigma }_{2}^{(1)}\\ {\sigma }_{2}^{(1)} & {\sigma }_{3}^{(1)}\end{array}\right)=\,\left(\begin{array}{cc}{a}_{1} & 0\\ {a}_{2} & {a}_{3}\end{array}\right){\left(\begin{array}{cc}{a}_{1} & 0\\ {a}_{2} & {a}_{3}\end{array}\right)}^{{\rm{T}}}$$

The free parameters *a*_1_, *a*_2_, *a*_3_, *σ*_1_^(2)^, *σ*_2_^(2)^ and *l* are estimated using maximum likelihood and automatic differentiation in Tensorflow^60,61^ using the BFGS algorithm. To improve convergence, we initialize *l* to the distance between centres of neighboring Visium spots.

This model allows us to infer which cell states are proximal in physical space and are likely to be migrating in the process of gradual differentiation in space.

For the spatial covariance model within StOrder workflow we only used a subset of our Visium data that corresponded to (1) decidua_and_villi_tips and (2) myometrium—because only these regions contained invading trophoblast cell states. For more details please see ‘Subsetting Visium data into anatomical regions with SpatialDE2’ in ‘Downstream analysis of 10x Genomics Visium data’ above. This helps to focus on the regions of the tissue that are relevant for the process of interest and is recommended to do in general if there are parts of the Visium data that do not contain cell states relevant to the process of interest.

Third, StOrder reconstructs connections between cell states by taking into account both the connectivity matrix (step 1) from single-cell transcriptomics data and the spatial covariance matrix (step 2) from the spatial data in the following way:

$${\beta }({\alpha }P+(1-{\alpha })S)+(1-{\beta })P\odot S$$

where *P* is the PAGA connectivity matrix, *S* is the spatial correlation matrix, *α* weights the contributions of *P* and *S* in the additive term, *β* weights the contributions of the additive and multiplicative terms, and ⊙ is the element-wise product. It then reconstructs the putative trajectory tree using the built-in PAGA functions.

The combined connectivity matrix based on both gene expression and spatial data with a range of weight parameters revealed the fully resolved invasion trajectory tree of the EVT with the correct topology (all connected cell state components, one branching point, no cycles, start at VCT-CCC population and two end points: eEVT and GC populations). The choice of ω parameter (contribution/weight of gene expression vs spatial part in the final matrix) in this last step depends on the goal of using this approach. In our case, we assumed: (1) the origin of EVT (VCT-CCC); (2) the end points of EVT (eEVT and GC); (3) the determination of a single branching point; and (4) the absence of cyclic trajectory. We therefore produced trajectory trees for 10,201 of (*α*,*β*) value pairs (from 0 to 1 with 0.01 increment step each) representative of different tree topologies corresponding to different ratios of gene expression vs spatial contribution. Out of the 10,201 tree structures we inspected, 3,574 trees represented the topology with the assumptions described above. These trajectories consistently assigned EVT-2 as the putative branching point. Tree structures with mainly gene expression-based connectivity values did not yield a branching point population we were looking for. Tree structures with mainly spatial based connectivities hindered the link between iEVT and GC populations, likely due to the large length scale of this invasion in space.

#### Limitations

Our approach assumes the gradual nature of gene expression changes accompanied by gradual migration of cells in space while they differentiate. Thus, it may not yield meaningful results in scenarios where this underlying assumption is violated. In addition, it is recommended that the user estimates the spatial scale at which the process of interest is taking place—whether in current Visium resolution the differentiation and migration is happening over the course of only a few spots or many more—this will change the initial values of l parameter and help the model fit the data better.

### Combined RNA and ATAC analysis using MEFISTO

#### Preprocessing of multiome data and training of the MEFISTO model

Gene expression (snRNA-seq) counts of the multiome data for donor P13 were normalized by total counts (scanpy.pp.normalize_per_cell(rna, counts_per_cell_after=1e4)) and log-transformed (pp.log1p(rna)). Highly variable gene features were then calculated (sc.pp.highly_variable_genes(rna, min_mean=0.0125, max_mean=3, min_disp=0.5)) and the subsetted object’s expression was scaled (sc.pp.scale(rna, max_value=10)).

Chromatin accessibility (scATAC-seq) counts of the multiome data for donor P13 were preprocessed using TF-IDF normalization (muon.atac.pp.tfidf(atac[key], scale_factor=1e4)). To select biologically meaningful highly variable peak features, ATAC counts were aggregated into pseodubulks by cell states and averaged, then variance of accessibility was calculated across these pseudobulks, and informative peak features were selected based on this measure (top 75th percentile (10,640) of peaks selected in total) as the peaks with highest variance. Finally, these data were scaled (sc.pp.scale(atac, max_value=10)).

Using the preprocessed RNA and ATAC data we used a pseudotime-aware dimensionality reduction method MEFISTO^30^ to extract major sources of variation from the RNA and ATAC data jointly and identify coordinated patterns along the invasion trajectory. As a proxy for the trophoblast invasion trajectory in the MEFISTO model we used 2-dimensional pseudotime coordinates based on a UMAP of the RNA data by calculating PCA (sc.tl.pca(rna, n_comps=8)), neighborhood graph (sc.pp.neighbors(rna)) and UMAP embedding (sc.tl.umap(rna)).

The MEFISTO model was trained using the following command within MUON (version 0.1.2) package interface:

muon.tl.mofa(mdata, outfile=’’,

use_obs = “union”,

smooth_covariate=[“UMAP1”, “UMAP2”],

use_float32=True)

We further excluded factor 5 from downstream analysis as a technical artefact due to its significant and high correlation (Spearman rank-order correlation coefficient 0.94 (over all cell states), *P* < 10^−308^, two-sided test) with the n_peaks_by_counts (number of ATAC peaks with at least 1 count in a nucleus) in ATAC view in all cell states (Supplementary Fig. 4k) and lack of smoothness along pseudotime (Supplementary Fig. 4j).

#### Defining groups of ATAC peak features

To further interpret ATAC features, we annotated them based on their genomic location using GenomicRanges package (version 1.42.0). In parallel, we used epigenetic data from^62^ to mark peak features in close proximity to trophoblast-specific enhancer features. To do so, we used peak files corresponding to H3K4me1, H3K27ac and H3K27me3 histone modifications marks for second trimester trophoblast samples (obtained from authors of aforementioned study upon request) to infer regions of the genome corresponding to active (H3K27ac + H3K27me3), primed (only H3K4me1) or repressed (H3K4me1 + H3K27me3) enhancers. This was done using bedtools (version 2.30.0) in the following way:

1. bedtools subtract -a H3K4me1_file.bed -b H3K27ac_file.bed > interm_file.bed bedtools subtract -a interm_file.bed -b H3K27me3_file.bed > primed_enhancers.bed To produce primed enhancers file
2. bedtools intersect -a H3K4me1_file.bed -b H3K27ac_file.bed > active_enhancers.bed To produce active enhancers file
3. bedtools intersect -a H3K4me1_file.bed -b H3K27me3_file.bed > repressed_enhancers.bed To produce repressed enhancers file

The enhancer files produced were then overlapped with peaks in ATAC analysis (bedtools intersect -a atac_peaks_file.bed -b enhancer_file.bed -wa) and any peaks having a >1-bp overlap with an enhancer feature were considered to be proximal to those features (done separately for active, primed and repressed enhancers).

#### Enrichment analysis of features in the MEFISTO model

Gene set enrichment analysis for gene features was performed based on the C5 category and the Biological Process subcategory from the MSigDB database (https://www.gsea-msigdb.org/gsea/msigdb) using GSEA functionality implemented in MOFA2 (run_enrichment command, MOFA2 version 1.3.5). This was done separately for negative and positive weights of each factor.

Peak group enrichment for peak features was performed using the same run_enrichment command in MOFA2 on peak groups defined as described above (Defining groups of ATAC peak features).

#### Transcription factor analysis using the MEFISTO model

To extract information about transcription factor binding motif enrichment in ATAC features of MEFISTO factors, we first performed enrichment analysis of peaks using GSEA functionality implemented in MOFA2 (run_enrichment command, MOFA2 version 1.3.5) on the peak-motif matrix produced by Signac package (version 1.5.0). Then, to identify which MEFISTO factors contribute the most to each transition of cell states along the invading trophoblast trajectory (inferred with StOrder), we trained logistic regression classifiers for each transition along the trajectory (overall for 6 transitions: VCT→VCT-CCC, VCT-CCC→EVT-1, EVT-1→EVT-2, EVT-2→iEVT, iEVT→GC, EVT-2→eEVT) on the matrix of factor values. For each transition the factor with the highest absolute coefficient separating the two cell states was selected, accounting for the sign of contribution in the logistic regression (positive or negative). If the top factor is contributing to a transition with a positive coefficient, transcription factor binding motifs coming from MEFISTO enrichment analysis of this factor’s top positive values are further considered in general transcription factor analysis as transcription factors upregulated upon this transition, whereas transcription factor binding motifs coming from MEFISTO enrichment analysis of this factor’s top negative values are further considered in general transcription factor analysis as transcription factors downregulated upon this transition. All of these transcription factor motifs are marked as having evidence from the MEFISTO factor relevant for this transition. Reverse procedure is applied in case if the top factor is contributing to a transition with a negative coefficient in the corresponding logistic regression model.

For more details please see the following notebook: https://github.com/ventolab/MFI/blob/main/2_inv_troph_trajectory_and_TFs/2-5_MEFISTO_analysis_inv_troph/S3_DEG_comparison_to_MEFISTO_factor_translation.ipynb

### Trophoblast trajectory inference analysis

To derive trophoblast pseudotime based on transcriptomic similarity, we used Slingshot v1.8.0. With Slingshot we fitted a cluster-based minimum spanning tree (MST) over the two-dimensional UMAP of P13 trophoblasts, and inferred the global lineage topology to assign cell states to lineages. Only donor P13 cells in the G1 phase of the cell cycle were included. To balance trophoblast state contributions, we downsampled each trophoblast state to account for up to 100 cells per state. VCT was assigned as the initial cell state (start.clus), while eEVT, SCT and GC were assigned as terminal states (end.clus). Slingshot fits simultaneous principle curves to smooth the MST and assigns a weight for each trophoblast cell in each lineage. Slingshot outputs lineage-specific pseudotimes and weights of assignment for each cell.

We next fitted a tradeSeq (v1.4.0) gene expression model (negative binomial generalized additive model) using the trajectory pseudotime and the weights computed with Slingshot (with nknots = 6). Next, we tested whether the gene expression is significantly changing along trophoblast pseudotime. For such a purpose, we used the statistical test implemented in the associationTest function, which tests the null hypothesis that all smoother coefficients are equal to each other. Genes with a *P* < 10^−6^ and mean logFC > 0.5 were selected as the main drivers of the trophoblast trajectory.

### Cell label transferring on trophoblast organoids

To transfer cell labels from donor P13 snRNA-seq in vivo trophoblast to the scRNA-seq TSC and PTO we trained two independent logistic regression models. The P13 dataset was downsampled to 500 cells per trophoblast state, except for GC and eEVT, which were discarded from the training due to their scarcely abundance. The common highly variable genes (1,695 genes for PTO and 1,565 for TSC), of the 4,000 selected per dataset, between the in vivo and each individual organoid dataset were selected as features for model training. The in vivo dataset was split into 80/20 training and test set, hyperparameters were explored employing a threefold cross-validation and scored employing the mean Matthews correlation coefficient of each fold. Top-ranked models were selected and assessed on the test set, with no significant differences found between them. Finally, the best model for each organoid dataset was employed to transfer cell labels from donor P13.

### Cell–cell communication analysis with CellPhoneDB

To retrieve interactions between invading trophoblast and other cell populations identified in our samples, we used the CellPhoneDB degs_analysis method^13,63^ (https://github.com/ventolab/CellphoneDB) described in ref. ^33^. In short, we retrieved the interacting pairs of ligands and receptors meeting the following requirements: (1) all the protein members were expressed in at least 10% of the cell type under consideration; and (2) at least one of the protein members in the ligand or the receptor was a DEG in an invading trophoblast subset (according to our analysis of differential expression, for details please see ‘Differential gene expression analysis’), with an adjusted *P*-value below 0.05 and logFC > 0.1. We further selected which cell states are spatially co-located in each microenvironment via visual inspection of cell2location deconvolution results for our Visium data. The analysis was done on an updated version of CellPhoneDB-database (v4.1) which includes novel intercellular interactions from refs. ^64^^,^^65^. Only bona fide manually curated interactions were considered in the analysis.

### Transcription factor analysis

To prioritize the transcription factors relevant for each invading trophoblast cell state or microenvironment, we integrate four types of measurements: (1) expression levels of the transcription factor and (2) the activity status of the transcription factor measured from (2a) the expression levels of their targets (described in ‘Transcription factor activities derived from scRNA-seq and snRNA-seq’) and/or (2b) the chromatin accessibility of their binding motifs (described in ‘Transcription factor motif activity analysis from scATAC–seq’) and/or (2c) evidence of the chromatin accessibility of their binding motifs in relevant factors from multimodal RNA-ATAC analysis (with MEFISTO). Plots in main figures include transcription factor meeting the following criteria: (1) transcription factor was differentially expressed, with adjusted *P*-value < 0.05) and/or (2) transcription factor was differentially active, with log_2_ fold change greater than 0.25 and adjusted *P*-value < 0.05 in at least one of the transcription factor activity measurements (2a or 2b).

#### Transcription factor differential expression from scRNA-seq and snRNA-seq

We compute differential expression using the procedure described in ‘Differential gene expression analysis’ and further subset resulting gene targets to transcription factors only based on the list of transcription factors provided by DoRothEA.

#### Transcription factor activities derived from scRNA-seq and snRNA-seq

We estimated protein-level activity for human transcription factor as a proxy of the combined expression levels of their targets. Target genes were retrieved from Dorothea^66^, an orthogonal collection of transcription factor targets compiled from a range of different sources. Next, we estimated transcription factor activities for each cell using Viper^67^, a GSEA-like approach, as implemented in the Dorothea R package and tutorial^68^ for the genes differentially expressed along the invading trophoblast trajectory (see ‘Differential gene expression analysis’).

#### Transcription factor motif activity analysis from scATAC–seq

Transcription factor motif activities were computed using chromVar^69^ v. 1.12.2 with positional weight matrices from JASPAR2018^70^, HOCOMOCOv10^71^, SwissRegulon^72^, HOMER^73^. chromVar returns a matrix with binding activity estimates of each transcription factor in each cell, which we used to test for differential transcription factor binding activity between trophoblast cell states with FindMarkers function in Seurat (default parameters) in the same way as described in ‘Differential gene expression analysis’ (backwards along invading trophoblast trajectory).

### Reporting summary

Further information on research design is available in the Nature Portfolio Reporting Summary linked to this article.

## Online content

Any methods, additional references, Nature Portfolio reporting summaries, source data, extended data, supplementary information, acknowledgements, peer review information; details of author contributions and competing interests; and statements of data and code availability are available at 10.1038/s41586-023-05869-0.

## Supplementary information

Supplementary Information This file contains a guide to Supplementary Tables 1–8 (tables supplied separately).

Reporting Summary

Supplementary Table 1 Sample metadata – see Supplementary Table Guide for details.

Supplementary Table 2 Quality control of samples for each 10X RNA library in our maternal-fetal interface atlas – see Supplementary Table Guide for details.

Supplementary Table 3 Annotation summary for each sample – see Supplementary Table Guide for details.

Supplementary Table 4 Variance explained (R2 column) in the MEFISTO model by each factor in each modality (RNA or ATAC) – see Supplementary Table Guide for details.

Supplementary Table 5 Differentially expressed genes (DEG) along trophoblast trajectories in P13 – see Supplementary Table Guide for details.

Supplementary Table 6 TF analysis along trophoblast trajectory – see Supplementary Table Guide for details.

Supplementary Table 7 Trophoblast interactions enriched by microenvironment (ME) using CellPhoneDB – see Supplementary Table Guide for details.

Supplementary Table 8 Probes used for multiplexed RNAscope smFISH.

## Data Availability

Open access datasets are available from ArrayExpress (www.ebi.ac.uk/arrayexpress), with accession numbers E-MTAB-12421 (scRNA-seq and snRNA-seq of primary tissue), E-MTAB-12595 (multiome snRNA-seq and snATAC-seq), E-MTAB-12698 (Visium), E-MTAB-12650 (scRNA-seq and snRNA-seq of PTOs). Managed-access datasets are available from EGA archive (https://ega-archive.org/) with accession numbers EGAD00001010037 (scRNA-seq and snRNA-seq of historical placental beds), EGAD00001010038 (multiome snRNA-seq and snATAC-seq of historical placental beds), EGAD00001010017 (scRNA-seq and snRNA-seq of TSCs). Image datasets are available at the EMBL–EBI BioImage Archive (https://www.ebi.ac.uk/biostudies) under accession number S-BIAD615. All datasets are public access. scRNA-seq and snRNA-seq datasets to reproduce UMAPs and dot plots can be accessed and downloaded through the web portals at https://www.reproductivecellatlas.org. The external scRNA-seq dataset of the first-trimester human decidual–placental interface is available from ArrayExpress (E-MTAB-6701).

## Code availability

All code used for data analysis is available at https://github.com/ventolab/MFI^74^.
