# Supplementary material for: Spatial multiomics map of trophoblast development in early pregnancy
Source: Nature. 2023 Mar 29;616(7955):143–51. doi: 10.1038/s41586-023-05869-0 (PMC10076224; doi:10.1038/s41586-023-05869-0)
Supplement: Supplementary file 1 — This file contains a guide to Supplementary Tables 1–8 (tables supplied separately). [file 41586_2023_5869_MOESM1_ESM.pdf]

nature portfolio

https://doi.org/10.1038/s41586-023-05869-0

Supplementary information

Spatial multiomics map of trophoblast development in early pregnancy

In the format provided by the authors and unedited

Supplementary Material

Supplementary Tables

Supplementary Table 1. Supplementary_Table_1.xlsx (separate file)
Metadata of samples. (A) 10X scRNA-seq libraries from human donors. **(B)** 10X snRNA-seq libraries from human donors. **(C)** 10X cell-coupled snRNA/ATAC-seq (multiome) libraries from human donors. **(D)** 10X Visium spatial transcriptomics libraries from human donors. **(E)** 10x libraries from organoid samples. **(F)** Metadata for human donors used in the study.
Sample id = 10x reaction; Donor = donor ID; Stage_PCW = post-conceptional weeks; TP = type of pregnancy termination; Media = organoid growth media: Model = organoid model. (Med: medical; Sur: surgical or Hys: hysterectomy)

Supplementary Table 2. Supplementary_Table_2.xlsx (separate file)
Quality control of samples for each 10X RNA library in our maternal-fetal interface atlas. (A) Summary statistics from 10X Cell Ranger 3.0.2 for scRNA-seq samples. **(B)** Summary statistics from 10X Cell Ranger 3.0.2 for snRNA-seq samples. **(C)** Summary statistics from 10X Cell Ranger ARC 1.0.1 for multiome samples. **(D)** Summary statistics from 10X Space Ranger 1.1.0 Visium spatial transcriptomics samples.

Supplementary Table 3. Supplementary_Table3.xlsx (separate file)
Annotation summary for each sample. Number of cells/nuclei (droplets) per coarse cell state in scRNA-seq, snRNA-seq and multiome samples of donors P13, P14, Hrv43 and all donors dataset.

Supplementary Table 4. Supplementary_Table4.xlsx (separate file)
Variance explained (R2 column) in the MEFISTO model by each factor in each modality (RNA or ATAC).

Supplementary Table 5. Supplementary_Table5.xlsx (separate file)
Differentially expressed genes (DEG) along trophoblast trajectories in P13. **(A)** TradeSeq test evaluating the association between average gene expression and Slingshot pseudotime (with Wald test using FDR values to rank genes). **(B)** DEG along the EVT trajectory in donor P13 in a retrograde manner (limma, FDR < 0.05, with Bonferroni correction for multiple hypotheses testing. **(C)** DEG along the EVT trajectory in all donors in a retrograde manner (limma, FDR < 0.05, with Bonferroni correction for multiple hypotheses testing)

Supplementary Table 6. Supplementary_Table5.xlsx (separate file)
TF analysis along trophoblast trajectory. Table containing the multiple TF measurements in the *in vivo* analysis used to prioritise TF relevant for trophoblast differentiation of all donors **(A)** and for donor P13 **(B)**. All tests are performed by comparing along the trophoblast differentiation axis.
Columns across the table indicate: cluster = cell type; TF = transcription factor; is_DE_limma = 1 or 0 (yes/no) if it is a differentially expressed TF (limma, FDR < 0.05, with Bonferroni correction for multiple hypotheses testing); is_DA_dorothea = 1 or 0 (yes/no) if it is a differentially activated TF (FDR < 0.05; Wilcoxon test); is_DA_chromVar = 1 or 0 (yes/no) if the TF binding motifs are differentially accessible (FDR < 0.05; Wilcoxon test). Finally, is_evidence indicates the number of supporting evidences found according to all measures.

Supplementary Table 7. Supplementary_Table7.xlsx (separate file)
Trophoblast interactions enriched by microenvironment (ME) using CellPhoneDB. **(A)** ME2 = cytotrophoblast cell column. **(B)** ME3 = Invasion front. **(C)** ME4 = Decidual/myometrial border. **(D)** ME5 = Spiral arteries.

Supplementary Table 8. Supplementary_Table8.xlsx (separate file)
Probes used for multiplexed RNAscope smFISH.
