# Supplementary material for: Spatial multiomics map of trophoblast development in early pregnancy
Source: Nature. 2023 Mar 29;616(7955):143–51. doi: 10.1038/s41586-023-05869-0 (PMC10076224; doi:10.1038/s41586-023-05869-0)
Supplement: Supplementary file 2 — Reporting Summary [file 41586_2023_5869_MOESM2_ESM.pdf]

nature portfolio

Corresponding author(s): Roser Vento-Tormo

Last updated by author(s): Feb 3, 2023

Reporting Summary

Nature Portfolio wishes to improve the reproducibility of the work that we publish. This form provides structure for consistency and transparency in reporting. For further information on Nature Portfolio policies, see our Editorial Policies and the Editorial Policy Checklist.

Statistics

For all statistical analyses, confirm that the following items are present in the figure legend, table legend, main text, or Methods section.

n/a	Confirmed	
☐	☒	The exact sample size (*n*) for each experimental group/condition, given as a discrete number and unit of measurement
☐	☒	A statement on whether measurements were taken from distinct samples or whether the same sample was measured repeatedly
☐	☒	The statistical test(s) used AND whether they are one- or two-sided<br>*Only common tests should be described solely by name; describe more complex techniques in the Methods section.*
☐	☒	A description of all covariates tested
☐	☒	A description of any assumptions or corrections, such as tests of normality and adjustment for multiple comparisons
☐	☒	A full description of the statistical parameters including central tendency (e.g. means) or other basic estimates (e.g. regression coefficient) AND variation (e.g. standard deviation) or associated estimates of uncertainty (e.g. confidence intervals)
☐	☒	For null hypothesis testing, the test statistic (e.g. *F*, *t*, *r*) with confidence intervals, effect sizes, degrees of freedom and *P* value noted<br>*Give P values as exact values whenever suitable.*
☐	☒	For Bayesian analysis, information on the choice of priors and Markov chain Monte Carlo settings
☒	☐	For hierarchical and complex designs, identification of the appropriate level for tests and full reporting of outcomes
☒	☐	Estimates of effect sizes (e.g. Cohen's *d*, Pearson's *r*), indicating how they were calculated

Our web collection on statistics for biologists contains articles on many of the points above.

Software and code

Policy information about availability of computer code

Data collection	No software was used for data collection
Data analysis	Alignment, QC and barcode calling of scRNA-seq and snRNA-seq data with Cell Ranger version 3.0.2 (10X Genomics)<br>Alignment, QC and barcode calling of multiomics GEX and ATAC-seq data with Cell Ranger-Arc version 1.0.1 (10X Genomics)<br>Peak calling and quantification of multiome ATAC-seq data with custom cellatac pipeline available at https://github.com/cellgeni/cellatac, revision 21-099<br>Alignment, QC and image processing of Visium spatial transcriptomics data with Space Ranger version 1.1.0 (10X Genomics)<br>Downstream analysis using custom code available at https://github.com/ventolab/MFI<br>Genotype-based deconvolution of sc/snRNA-seq with Souporcell version 2.4.0<br>Analysis of sc/snRNA-seq with scanpy (version 1.7.1)<br>Differential gene expression analysis with limma version 3.46.0 and edgeR version 3.32.1<br>Analysis of multiome GEX and ATAC-seq data with the following packages: Signac version 0.2.5 (https://satijalab.org/signac/), MUON version 0.1.2, GenomicRanges version 1.42.0, bedtools version 2.30.0, MOFA2 version 1.3.5<br>Differential gene expression analysis with limma version 3.46.0, edgeR version 3.32.1 and tradeSeq version 1.4.0

For manuscripts utilizing custom algorithms or software that are central to the research but not yet described in published literature, software must be made available to editors and reviewers. We strongly encourage code deposition in a community repository (e.g. GitHub). See the Nature Portfolio guidelines for submitting code & software for further information.

Data

Policy information about availability of data

All manuscripts must include a data availability statement. This statement should provide the following information, where applicable:

- Accession codes, unique identifiers, or web links for publicly available datasets
- A description of any restrictions on data availability
- For clinical datasets or third party data, please ensure that the statement adheres to our policy

Data availability: Open access datasets are available from ArrayExpress (www.ebi.ac.uk/arrayexpress), with accession numbers E-MTAB-12421 (scRNA-seq/snRNA-seq of primary tissue), E-MTAB-12595 (multiome snRNA-snATAC-seq), E-MTAB-12698 (visium), E-MTAB-12650 (scRNAseq/snRNA-seq of primary trophoblast organoids). Managed access datasets are available from EGA archive (https://ega-archive.org/) with accession number EGAD00001010037 (scRNA-seq/snRNA-seq of historical placental beds), EGAD00001010038 (multiome snRNA-snATAC-seq of historical placental beds), EGAD00001010017 (scRNAseq/snRNA-seq of trophoblast stem cell). Image datasets are available at the EMBL-EBI BioImage Archive (www.ebi.ac.uk/biostudies) under accession number S-BIAD615. All datasets are public access. scRNA-seq and snRNA-seq datasets to reproduce UMAPs and dot plots can be accessed and downloaded through the web portals www.reproductivecellatlas.org. External scRNA-seq dataset of the first-trimester human decidual-placental interface is available from ArrayExpress (E-MTAB-6701).

Field-specific reporting

Please select the one below that is the best fit for your research. If you are not sure, read the appropriate sections before making your selection.

☒ Life sciences ☐ Behavioural & social sciences ☐ Ecological, evolutionary & environmental sciences

For a reference copy of the document with all sections, see nature.com/documents/nr-reporting-summary-flat.pdf

Life sciences study design

All studies must disclose on these points even when the disclosure is negative.

Sample size	We collected primary placental tissues from 11 individuals. This cohort is equal or larger than previous single-cell transcriptomic atlases of placental tissues in humans (PMID: 30429548, PMID: 30402542), and should be sufficient to capture the main cell types and states in the tissue. In addition, novel subsets defined transcriptomically in our dataset (e.g. endovascular extravillous trophoblast) have been validated using orthogonal methods (e.g. spatial transcriptomics, immunohistochemistry)
Data exclusions	No data were excluded from the analyses
Replication	For single cell and nuclei transcriptomics replicates were considered for the majority of the donors. For donor P13 (6 libraries), donor P14 (4 libraries), donor P34 (1 library), donor Hrv43 (4 libraries), donor Hrv46 (3 libraries), donor H2 (2 libraries), donors H7 + H9 (pooled, 3 libraries), donor Hrv98 (1 library), donor Hrv99 (1 library), donor Hrv100 (1 library). Analysis of technical replicates revealed the same populations.<br><br>For single nuclei RNA and ATAC seq (snRNA-seq/snATAC-seq), duplicates were considered in the majority of cases. For donor P13 (2 libraries), donor P14 (1 library), donor Hrv43 (3 libraries).Analysis of technical replicates revealed the same placental populations.<br><br>For spatial transcriptomics, we included five replicates (four consecutive tissue slides and one slide from another tissue block) for donor P13 and two replicates (two consecutive slides) for donor P14. For high-resolution imaging using RNAScope probes, we performed the analysis on at least two slides from distinct individuals, and this is indicated in the figure legends.<br><br>For primary trophoblast organoids, experiments were performed on organoids derived from six distinct donors. For trophoblast stem cells, two distinct donors were used. Differences between individuals are expanded in the text.
Randomization	This is not relevant for this study as we are not comparing any disease group.
Blinding	This is not relevant for this study as we are not comparing any disease group.

Reporting for specific materials, systems and methods

We require information from authors about some types of materials, experimental systems and methods used in many studies. Here, indicate whether each material, system or method listed is relevant to your study. If you are not sure if a list item applies to your research, read the appropriate section before selecting a response.

Materials & experimental systems

n/a	Involved in the study	
☐	☒	Antibodies
☐	☒	Eukaryotic cell lines
☒	☐	Palaeontology and archaeology
☒	☐	Animals and other organisms
☐	☒	Human research participants
☒	☐	Clinical data
☒	☐	Dual use research of concern

Methods

n/a	Involved in the study	
☒	☐	ChIP-seq
☒	☐	Flow cytometry
☒	☐	MRI-based neuroimaging

Antibodies

Antibodies used	NCAM1<br>Company: Cell Signaling Technology<br>Cat. #: 3576S<br>lot #: 9<br>clonality: monoclonal<br>clone number: 123C3<br>host: mouse<br>isotype: IgG1<br>dilution rate: 1:50<br>buffer: citrate
Validation	NCAM1 . Immunohistochemistry on paraffin-embedded tissue (website).

Eukaryotic cell lines

Policy information about cell lines

Cell line source(s)	BTS5 and BTS11 derived from Okae 2017 Cell Stem Cell (DOI: 10.1016/j.stem.2017.11.004)
Authentication	None in-house, lines were imported into UK directly from Dr. Okae in Japan
Mycoplasma contamination	Testing was done at the Gurdon Institute before the lines were transferred to Sanger using the Lonza MycoAlert™ mycoplasma detection kit. Luminescence levels were not above threshold compared to an internal negative control.
Commonly misidentified lines<br>(See ICLAC register)	None

Human research participants

Policy information about studies involving human research participants

Population characteristics	All samples were between 4-13 post conceptional weeks.
Recruitment	All tissue samples used for this study were obtained with written informed consent from all participants in accordance with the guidelines in The Declaration of Helsinki 2000. An exception is Placental/decidual blocks (P13, P14 and P34) that were collected prior to 1 September 2006 and consent for research use was not obtained. These samples are considered 'Existing Holdings' under the Human Tissue Act and as such were able to be used in this project.
Ethics oversight	Placental and decidual samples used for the in vivo and in vitro profiling were obtained from elective terminations from:<br>- The MRC and Wellcome-funded Human Developmental Biology Resource (HDBR, http:// www.hdbr.org), with appropriate maternal written consent and approval from the Fulham Research Ethics Committee (REC reference 18/LO/0822) and Newcastle & North Tyneside 1 Research Ethics Committee (REC reference 18/NE/0290). The HDBR is regulated by the UK Human Tissue Authority (HTA; www.hta.gov.uk) and operates in accordance with the relevant HTA Codes of Practice.<br><br>- Addenbooke's Hospital (Cambridge) under ethical approval from the Cambridge Local Research Ethics Committee (04/Q0108/23), which is incorporated into The overarching ethics permission given to the Centre for Trophoblast Research biobank for the "Biology of the Human Uterus in Pregnancy and Disease Tissue Bank" at the University of Cambridge under ethical approval from the East of England-Cambridge Central Research Ethics Committee (17/EE/0151) and from the London-Hampstead Research Ethics Committee (20/LO/0115).

Note that full information on the approval of the study protocol must also be provided in the manuscript.
